# High resolution immunoinformatic profiling of Zonula occludens toxin reveals a conserved multiepitope vaccine candidate in *Acinetobacter baumannii*

**DOI:** 10.3389/fimmu.2026.1835523

**Published:** 2026-06-04

**Authors:** Payam Benyamini

**Affiliations:** Department of Biomedical Sciences, College of Medicine, Charles R. Drew University, Los Angeles, CA, United States

**Keywords:** *Acinetobacter baumannii*, epitope-based vaccine design, immunoinformatics analysis, reverse vaccinology, Zonula occludens toxin (Zot)

## Abstract

*Acinetobacter baumannii* is a multidrug-resistant (MDR) nosocomial pathogen associated with exceptionally high mortality, underscoring the urgent need for immune-based strategies that operate independently of antibiotic susceptibility. The Zonula occludens toxin (Zot), a barrier-disrupting virulence factor embedded within mobile genetic elements, has recently been proposed as a conserved component of clinically relevant *A. baumannii* lineages, however, its immunological architecture remains poorly defined. Here, we applied an integrated immunoinformatic and evolutionary framework to evaluate Zot as a target for adaptive immune recognition and immunotherapeutic intervention. Comparative analysis of 62 full-length Zot homologs revealed structured sequence diversity superimposed on near-complete alignment continuity, consistent with structural constraint maintaining protein integrity. Structural modeling demonstrated that deep sequence divergence between historical and contemporary isolates does not disrupt the overall Zot fold, indicating conservation of antigenic architecture. High-resolution epitope mapping identified multiple potential B-cell–accessible regions and dense clusters of conserved MHC Class I and Class II binding motifs, particularly within a compact C-terminal domain exhibiting strong sequence and structural constraint. Notably, predicted T-cell epitopes remained resilient across divergent strains, consistent with conserved MHC anchor residue architecture. Together, these findings suggest that Zot may represent a structurally constrained immunological target with features consistent with both humoral and cell-mediated immune engagement across genetically diverse *A. baumannii* backgrounds. This immune-centric characterization provides a mechanistic foundation for immunotherapeutic targeting of Zot and supports its prioritization for further experimental evaluation in vaccine development pipelines targeting MDR *A. baumannii*.

## Introduction

Toxins are among the most potent virulence factors employed by pathogenic microorganisms to manipulate host physiology and promote infection (1, 2). These specialized proteins or peptides can directly target cell membranes (3, 4), disrupt essential cellular processes such as protein synthesis (5, 6), affect neuronal function (7), compromise barrier integrity (8, 9), suppress immune responses (10, 11), alter cytoskeleton (12, 13) and induce cell death (14, 15). By targeting specific host structures or signaling pathways, toxins facilitate bacterial colonization, persistence, and dissemination. In many infectious diseases, toxin production is a key determinant of clinical outcome. This is underscored by experimental evidence demonstrating that administration of even minute quantities of purified bacterial toxins can reproduce hallmark features of infection, including inflammation, tissue damage, and systemic symptoms (16, 17). While the roles of well-characterized toxins—such as tetanus (7), diphtheria (18), clostridium (14, 19), pertussis (20), cholera (21), and botulinum toxins (7)—are well understood, the functions of several newly identified or less-studied toxins remain poorly defined, particularly in clinical context. One such example is the Zonula occludens toxin (Zot), originally identified in *Vibrio cholerae (*8, 22), and recently associated with numerous Gram-negative bacterial (GNB) pathogens of significant clinical concern (23, 24).

Epithelial tissues form tightly organized cellular barriers that line both internal and external surfaces of the body and perform essential physiological roles (25–28). A key feature of epithelial integrity is the presence of tight junctions (TJs)—complex subcellular structures composed of cytosolic and transmembrane proteins that seal adjacent epithelial cells and preserve barrier function (29–33). Although epithelial tissues act as robust barriers to microbial invasion, various pathogenic GNB, other than *V. cholerae*, also encode Zot homologs capable of modulating these junctions. However, the presence and functional role of Zot in other GNB, including *Acinetobacter baumannii*, remain incompletely defined and are the subject of ongoing investigation (23, 24). Recent studies, including work from our group, have proposed that Zot may function as a virulence-associated factor in *A. baumannii* (23*)*.

Zot is a 45 kDa membrane protein with Zot-like homologs identified in multiple GNB, including *V. cholerae, A. baumannii, Pseudomonas aeruginosa, Neisseria gonorrhoeae, Neisseria meningitidis, Klebsiella pneumoniae, Escherichia coli, Salmonella enterica, and Yersinia pestis* species (23, 24). However, functional characterization outside of *Vibrio* species remains limited. Zot is a host-mimicking toxin that exerts its effects by commandeering TJs in both epithelial and endothelial cells, which are essential for preserving barrier integrity between adjacent cells in tissues like the intestinal lining and the blood-brain barrier (8, 34, 35). Zot interacts with specific surface receptors on epithelial cells and activates intracellular signaling pathways that remodel the cytoskeleton and disturb TJ-associated proteins such as occludin, claudins, and ZO-1 (8, 22, 36, 37). This disruption weakens intercellular adhesion, increases paracellular permeability, impairs overall barrier function, and facilitates microbial translocation and systemic spread of bacterial pathogens and their toxins. The widespread presence and sequence conservation of Zot-like proteins among diverse GNB raise the possibility of a conserved biological role, although direct experimental evidence supporting a universal virulence function across species remains limited (24). Thus, it may serve as a potential candidate for further evaluation in vaccine development for immunotherapeutic purposes.

*A. baumannii* is a strict aerobic, non-motile, non-fermentative Gram-negative bacillus that has emerged over the past two decades as a leading cause of severe ICU-acquired infections, particularly ventilator-associated pneumonia (VAP) and bloodstream infection (BSI) (38–45). Its clinical success is driven by environmental persistence, including survival for months on hospital equipment and skin (46–49), extensive biofilm formation (46), and the ability to rapidly acquire antimicrobial resistance determinants (50). The organism has a genome of approximately 4 Mb with ~40% GC content (51), containing genomic islands (52, 53), bacteriophage elements (54), and plasmids that facilitate horizontal gene transfer and adaptation (51). A*. baumannii* virulence is supported by well-established factors such as acinetobactin-mediated iron acquisition (55), outer membrane vesicles (56, 57), enzymatic activity (lipases and proteases) (58, 59), and immune evasion mechanisms (60, 61). Clinically, *A. baumannii* causes a wide spectrum of infections including VAP (40, 44), bacteremia/sepsis (41, 43), wound and burn infections (62, 63), meningitis (50, 64), and urinary tract infections (65, 66). Treatment is complicated by multidrug resistance (MDR) and carbapenem resistance mediated by β-lactamases (including OXA-type) (67, 68), efflux pumps (69), altered penicillin-binding proteins (70, 71), and permeability changes (72–74), often delaying effective therapy and contributing to poor outcomes.

Comparative analyses across major nosocomial pathogens demonstrate that *A. baumannii* exhibits the highest lethality, with synthesis of 30-day mortality across multiple cohorts revealing consistently severe outcomes. In extensively drug-resistant (XDR) bacteremia, mortality reaches approximately 73%, representing the highest observed rate (75). In a broader cohort including VAP, bacteremia, urinary tract infections, and wound infections, mortality is lower but still substantial at ~42% (76). In VAP-specific studies, mortality ranges from 53–55%, increasing to ~60% in infections caused by high-cytotoxicity strains (77), while drug-resistant VAP cohorts report ~55% mortality at 28–30 days (78). Across studies, MDR *A. baumannii* infections demonstrate an overall ~56% 30-day mortality, exceeding that of other major nosocomial pathogens. These findings underscore the disproportionate clinical burden of *A. baumannii* and highlight the urgent need for therapeutic strategies that operate independently of antibiotic susceptibility, including immunotherapy and vaccine development.

Due to the urgent need for alternative therapies, reverse vaccinology provides a powerful framework for identifying conserved and immunogenic antigens directly from genomic data using computational approaches (79). Given the assumption that a pathogen’s proteome can be expressed at any time based on its genome, making it available for scientists without the need for cultivation, the idea includes the use of numerous advanced bioinformatics tools to identify promising candidates for vaccine development. This new bioinformatic driven methodology is known as “reverse vaccinology” (80). Amid scientific progress, these new sophisticated *in silico* reverse vaccinology methods have grown enormously and have enabled extensive genome and proteome mining, resulting in the prediction of candidate antigens with potential relevance for downstream experimental validation (81–84).

Given the pervasive evolutionary distribution, structural conservation, and potent barrier-disrupting capacity of Zot among clinically significant GNB pathogens, identifying and characterizing such virulence-associated proteins represents a crucial step toward developing targeted immunotherapies against *A. baumannii*. In this study, we apply an integrated immunoinformatic and evolutionary framework to evaluate Zot as a candidate immunological target. By combining sequence conservation, structural modeling and high resolution epitope prediction, we aim to define whether Zot contains conserved immunogenic features suitable for vaccine development against MDR *A. baumannii*.

## Results

**Figure 1:**
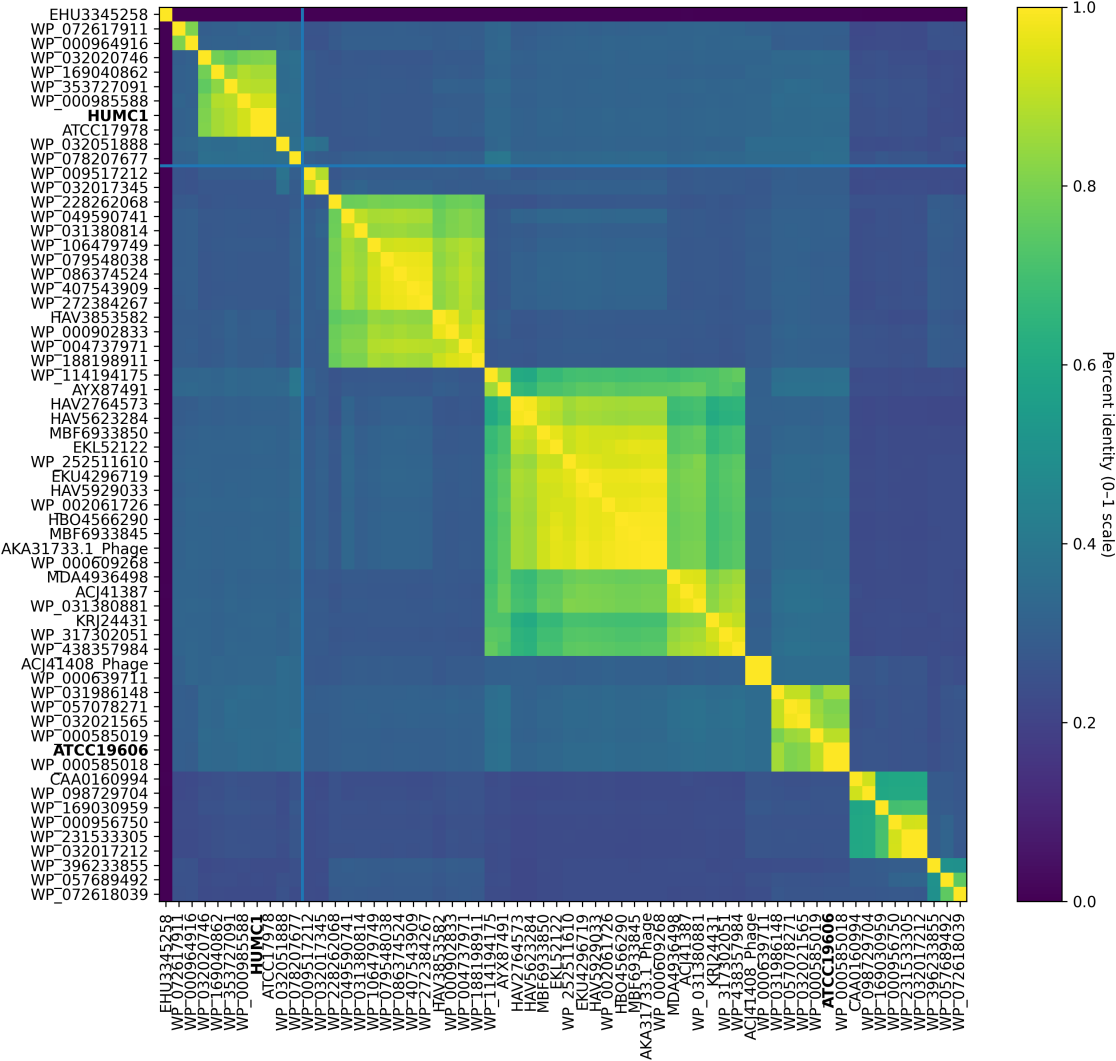
Conservation landscape of Zot across 62 *A. baumannii* strains. Global pairwise amino-acid identity values for full-length Zot proteins were computed and displayed as a clustered heatmap. Sequences were grouped according to higher similarity to either HUMC1 or ATCC 19606 and clustered within each group. Two major similarity lineages emerged: a highly conserved HUMC1-like cluster (≥80–100% identity in many strains, including 100% identity with ATCC 17978) and a more divergent ATCC 19606-like cluster (~25–50% identity to HUMC1). HUMC1 and ATCC 19606 share ~34% identity. Conserved regions within the HUMC1-like cluster overlapped strongly with predicted B- and T-cell epitopes, supporting their candidacy for broad-coverage antigen design.

Global amino-acid identity clustering reveals two major Zot similarity groups with conserved epitope-rich regions. To evaluate antigenic conservation of Zot across the *A. baumannii* species complex in the context of reverse vaccinology, we generated an all-vs-all global amino-acid identity matrix from 62 full-length Zot proteins. Pairwise identity values ranged from ~25–100%, indicating substantial but structured diversity across strains (**Figure 1**). To anchor the analysis to clinically relevant lineages, each sequence was assigned to the group showing higher global identity to either the contemporary hypervirulent bloodstream isolate HUMC1 or the historical reference strain ATCC 19606 (23). Hierarchical clustering was then performed within each group. This strategy allowed us to determine whether antigenic conservation is preferentially retained among modern clinical isolates compared to older laboratory lineages.

The resulting heatmap revealed two major similarity clusters rather than a continuum of divergence. A large subset of strains clustered tightly with HUMC1 and exhibited ≥80–100% amino-acid identity, including complete identity between HUMC1 and ATCC 17978 (**Figure 1**). In contrast, a second group of Zot sequences clustered more closely with ATCC 19606 and showed substantially lower identity to HUMC1 (~25–50%), although internal conservation within this group remained relatively high. HUMC1 and ATCC 19606 themselves shared only ~34% identity, reflecting deep evolutionary separation between the two major lineages (**Figure 1**). Thus, Zot is neither universally conserved nor randomly variable, but segregates into two evolutionarily coherent conservation groups that each retain internal sequence stability.

The predominance of highly conserved HUMC1-like sequences among recent clinical isolates suggests that Zot retains core structural or functional constraints in circulating *A. baumannii* populations. This pattern is consistent with purifying selection acting on at least part of the protein and indicates that Zot conservation may reflect underlying structural or functional constraints, although the biological role of Zot in *A. baumannii* remains to be experimentally established (23). Such constraint is a key feature sought in reverse-vaccinology pipelines, as it increases the likelihood that immune recognition is preserved across diverse strains.

We next examined how this conservation landscape intersects with predicted immunogenicity. B-cell epitope prediction (Bepipred 2.0) and MHC Class I/II T-cell epitope mapping (NetMHCpan and NetMHCIIpan) revealed multiple high-confidence epitopes located within the most strongly conserved regions of the HUMC1-like cluster, including regions showing ≥80–100% identity across strains. Several epitopes also remained at least partially conserved within ATCC 19606-like sequences despite much lower global identity. These findings indicate that immune-visible regions frequently overlap the most structurally constrained regions of Zot, highlighting them as candidates for further experimental evaluation in broad-coverage vaccine design.

From a reverse-vaccinology standpoint, the contrasting degrees of conservation between HUMC1 and ATCC 19606 Zot homologs provide a powerful framework for rational antigen and epitope selection. The extreme conservation observed between HUMC1 and multiple contemporary nosocomial isolates—including complete identity with ATCC 17978—suggests that a substantial portion of the Zot protein, likely corresponding to its structural core, represents a “high-value” antigenic substrate predicted to contain features consistent with cross-protective immune recognition. At the same time, the markedly lower identity between HUMC1 and ATCC 19606 (~34%) provides a natural stress-test for epitope robustness: epitopes that remain conserved despite deep overall divergence are likely to represent structurally essential, functionally irreplaceable regions and therefore represent high-priority candidates for experimental validation in vaccine design frameworks.

In this framework, even the divergence of ATCC 19606 becomes biologically informative. Regions that drift extensively between the two clusters can be deprioritized as unstable vaccine targets, whereas residues that resist substitution across both conserved groups likely encode evolutionary anchor points necessary for maintaining fold integrity or function. Notably, several of the strongest-predicted B- and T-cell epitopes mapped to these conserved regions, reinforcing the possibility that immunogenic and structurally constrained domains overlap. Such “deeply conserved” epitopes, which persist across hypervirulent modern isolates and evolutionarily distant strains, are particularly desirable because they are inherently less permissive to antigenic escape.

Together, these data indicate that Zot diversity is structured, constrained, and immunologically informative. The HUMC1–ATCC 19606 comparative framework allows discrimination between transient, strain-restricted residues and stable, evolutionarily locked-in epitopes, enabling the rational prioritization of Zot-derived vaccine targets that focus on the immutable backbone of the protein rather than its flexible periphery. This integrated approach — combining identity clustering with epitope prediction — provides a rigorous, sequence-driven foundation for advancing Zot as a candidate antigen for *A. baumannii* vaccine development.

Proteome-based alignments of Zot reveal two extended conserved regions. Multiple sequence alignment (MSA) tools were employed to perform a comparative proteomic analysis of Zot sequences derived from 58 distinct *A. baumannii* strains and four corresponding phages. These tools are indispensable for predicting structural and functional properties of proteins and for identifying evolutionarily conserved motifs that may represent robust vaccine candidates (85, 86). The principle underlying this approach is that regions of high conservation across genetically diverse strains are less likely to tolerate mutations without loss of function, making them ideal targets for eliciting broad-spectrum immune protection.

**Figure 2:**
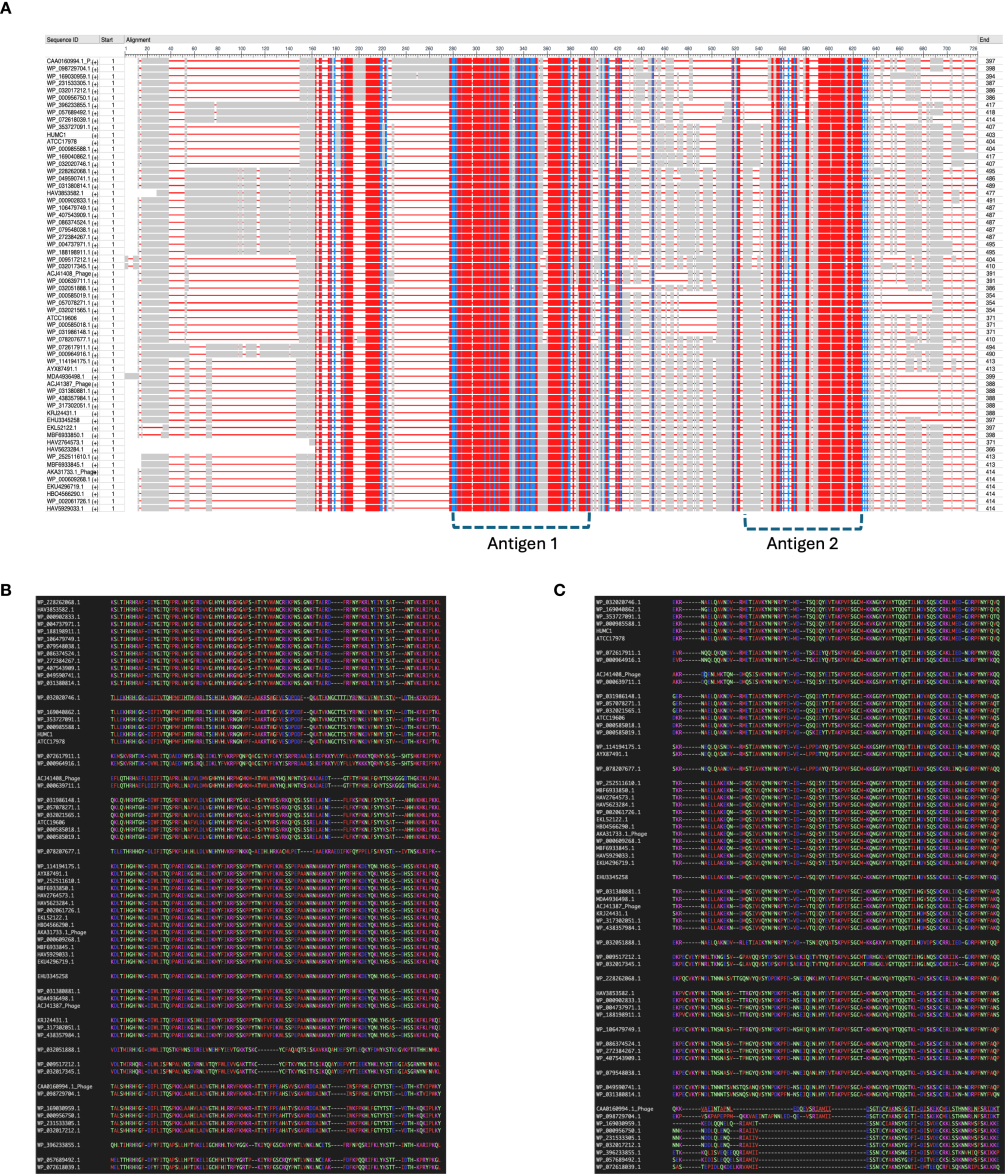
Multiple sequence alignment and conservation analysis of the Zot protein across a variety of *A. baumannii* strains and associated phages identifies two conserved potential antigens. **(A)** Multiple sequence alignment of Zot proteins from 58 *A. baumannii* clinical isolates and four associated phages highlights regions of evolutionary conservation across genetically diverse strains. Highly conserved residues are indicated in red. Two prominent conserved segments (~90–101 amino acids each) are identified: an N-terminal region (Antigen 1) and a C-terminal region (Antigen 2). Based on the HUMC1 Zot sequence, these correspond to residues Thr92–Leu192 and Glu266–Gln355, respectively. The strong conservation of these domains suggests essential functional roles and supports their prioritization as multi-strain antigenic targets for vaccine development. **(B, C)** Multiple sequence alignment of the two newly identified Zot antigens across diverse *A. baumannii* clinical isolates and associated phages reveals clear stratification into distinct sequence-homology subgroups. **(B)** shows the sequence and alignment of Antigen 1, in which strains segregate into 17 subgroups of varying sizes, reflecting discrete patterns of sequence conservation and divergence. Panel **(C)** depicts the alignment of Antigen 2, which resolves into 18 subgroups, including one subgroup comprising strains with minimal homology to the remaining sequences. Notably, Antigen 2 includes a subgroup containing both HUMC1 and ATCC 17978 reference strains. Together, these alignments demonstrate that while both antigens are broadly conserved, they exhibit structured intra-species diversity, consistent with evolutionary branching across strains and phages. This subgroup organization informs antigen selection by highlighting conserved cores shared across multiple lineages while capturing sequence variants relevant for broad spectrum vaccine design. Amino acid color alignments are based according to similarities in amino acid properties. [green, polar uncharged; magenta, positively charged; red, hydrophobic; blue, negatively charged].

Proteomic sequence alignments of Zot reveal the presence of two remarkably conserved stretches of approximately ~90 – 101 amino acids long, for both antigens, that are shared among a wide array of *A. baumannii* genetic variants (**Figure 2**). These conserved regions reflect a strong evolutionary constraint, suggesting that they play a fundamental role in maintaining the structural integrity or functional activity of the Zot protein. The identification of such regions is critical, as conserved amino acid motifs often correspond to biologically important domains that can serve as promising targets for vaccine development.

Our alignment results reveal multiple highly conserved stretches scattered throughout different regions of the Zot protein, as indicated by red coloration in the conservation map (**Figure 2**). Notably, these conserved segments map to the N-terminal region (Antigen 1; residues Thr92-Leu192) and the C-terminal region (Antigen 2; residues Glu266-Gln355) in the HUMC1 reference sequence. The high degree of conservation within these domains suggests essential structural or functional roles and supports their prioritization as candidate antigenic regions for vaccine development.

**Figure 3:**
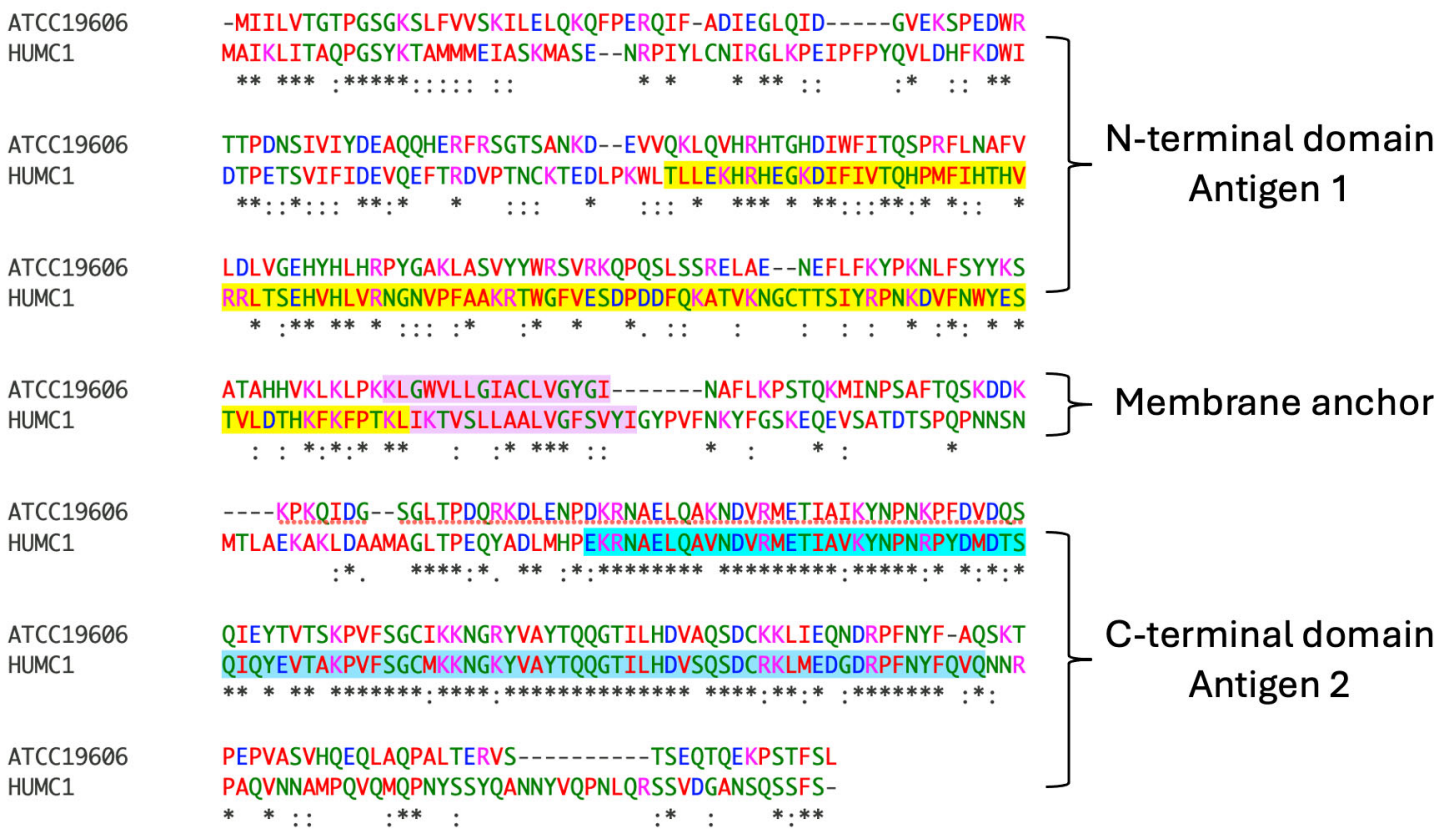
Domain organization and sequence conservation patterns of Antigen 1 and Antigen 2 in *A. baumannii* reveal a highly conserved C-terminus. Schematic representation of the membrane topology and domain architecture showing that Antigen 1 (amino acids **~**91 – 192) maps to the N-terminal region, whereas Antigen 2 (amino acids **~**265 – 355) localizes to the C-terminal region of the protein. These two antigenic domains are separated by a single predicted transmembrane α-helix (amino acids ~193 – 209), which anchors the protein in the bacterial outer membrane and spatially segregates the extracellular/periplasmic C-terminal domain from the cytosolic N-terminal domain. Comparative BLASTp analysis between ATCC 19606 and HUMC1 strains demonstrates high sequence heterogeneity and broad variability within the N-terminal domain (Antigen 1), consistent with rapid evolutionary drift and potential immune evasion mechanisms. In contrast, the C-terminal domain containing Antigen 2 exhibits strong sequence conservation, with percent identity values clustering tightly in the 70–95% range across strains, indicating functional constraints and evolutionary stability. These findings highlight Antigen 2 as a consistently conserved antigenic region, while Antigen 1 represents a highly variable sequence segment with limited cross-strain conservation.

Comparative alignment of HUMC1 and ATCC 19606 Zot reveals deep sequence divergence superimposed on full-length structural conservation. To assess the degree of conservation between Zot proteins from contemporary and historical *A. baumannii* lineages, we performed a pairwise BLASTp alignment using the HUMC1 full-length Zot protein as the reference query against the ATCC 19606 proteome. A single, high-confidence ATCC 19606 Zot homolog was identified, exhibiting nearly complete end-to-end alignment with the HUMC1 protein (373 aligned residues spanning positions 3–373 in HUMC1 and 2–350 in ATCC 19606) (**Figure 3**). Despite the extensive coverage across the full protein length, amino acid identity was limited to 38.9%, with 55.5% of positions classified as conservative substitutions, and only a small number of gap-containing regions observed (**Figure 3**). The alignment was associated with an E-value of 7.17 × 10^-82^ and a bit score of 243, confirming that the two proteins are unequivocally homologous despite substantial primary-sequence divergence. These findings indicate that HUMC1 and ATCC 19606 encode orthologous full-length Zot proteins that have retained a shared ancestral backbone architecture while undergoing extensive residue-level turnover.

The most striking feature of this comparison is the combination of deep divergence and near-complete alignment continuity. The preservation of full-length alignability strongly implies that the global domain architecture of Zot — including the N-terminal transmembrane-associated region and the C-terminal globular toxicogenic domain — has been retained across strains separated by substantial evolutionary distance. At the same time, the relatively modest primary-sequence identity (~40%) suggests that a large fraction of amino acids has been replaced or chemically modified over time. The high proportion of conservative substitutions suggests that these replacements tend to preserve biochemical character rather than introducing disruptive alterations, consistent with purifying selection acting to stabilize core structural features while permitting surface-level drift (87–89).

Closer inspection of the alignment reveals that this constraint is not distributed uniformly across the protein. The C-terminal domain — corresponding to our defined Antigen 2 region — is markedly more conserved at the primary-sequence level than the N-terminal domain containing Antigen 1. Within Antigen 2, a higher proportion of residues are either identical or conservatively substituted, and alignment gaps are rare, indicating that both the linear sequence and local residue chemistry are maintained to a greater extent in this region. This observation is consistent with the predicted structural role of the C-terminal domain as the principal folded globular toxin module, where disruption of core residues would be expected to compromise stability or function (8, 36, 90). By contrast, the N-terminal region — including Antigen 1 — shows substantially greater residue-level plasticity, with a higher incidence of substitutions and local variability. This asymmetry suggests that Antigen 2 is subject to stronger purifying selection, preserving functionally critical residues and overall fold integrity, whereas Antigen 1 tolerates a broader spectrum of evolutionary change, potentially reflecting roles in host interaction, secretion, localization, or immune evasion. Thus, the HUMC1–ATCC 19606 alignment supports a model of domain-specific evolutionary constraint in Zot, in which a rigid C-terminal toxin scaffold anchors functionality while the N-terminal region evolves more freely.

From an evolutionary perspective, this profile is highly informative. Full-length alignment across hundreds of residues at ~40% identity places Zot within the “twilight zone” of protein evolution, where structural conservation persists after extensive sequence divergence (91, 92). This pattern is characteristic of proteins experiencing long-term evolutionary stability of function, in which the overall fold and key physiochemical features are preserved by purifying selection, while surface-exposed or immune-facing residues diversify (87, 93–95). In the context of *A. baumannii*, where Zot is embedded within mobile genetic elements and prophage islands (96, 97), such a pattern suggests that Zot is not simply a passive cargo gene but rather a functionally maintained virulence module whose core activity remains advantageous despite genomic flux. The presence of a full-length, structurally coherent Zot in both the hypervirulent HUMC1 isolate and the historical ATCC 19606 reference strain further indicates that Zot is a stable and persistent component of the *A. baumannii* accessory genome, retained over deep evolutionary time.

These evolutionary dynamics have direct implications for reverse-vaccinology-based antigen discovery. For B-cell-mediated immunity, epitope recognition depends strongly on local residue identity and exposed surface chemistry (98, 99). The preferential conservation of Antigen 2 suggests that this C-terminal region is more likely to harbor cross-protective antibody epitopes that remain stable across diverse *A. baumannii* lineages. In contrast, Antigen 1, located within a more variable N-terminal context, may present isolate-specific epitopes, offering opportunities for strain-targeted serological responses but less suited for universal vaccine coverage. This domain-level distinction provides a rational framework for epitope prioritization, in which structurally constrained and evolutionarily conserved regions of Antigen 2 are favored for broad-spectrum vaccine design, while Antigen 1 is recognized as a potential contributor to antigenic diversity.

In contrast, T-cell epitopes are expected to be more resilient to the observed inter-strain variability. Because MHC binding relies primarily on short peptide motifs and conserved anchor residues rather than complete identity (100–102), the presence of >55% conservative substitutions across the alignment implies that many high-affinity MHC-I and MHC-II epitopes derived from HUMC1 Zot are predicted to remain immunologically recognizable in the ATCC 19606 ortholog. Thus, even in the setting of widespread sequence drift, cross-strain T-cell recognition is likely to persist, supporting the feasibility of designing multi-epitope constructs that warrant experimental testing for cellular immunogenicity across genetically diverse *A. baumannii* backgrounds. In this way, Zot exemplifies a broader principle of mobile-element-encoded toxins: they evolve under asymmetric constraint, preserving functionally essential structural scaffolds — most prominently within the conserved Antigen 2 domain — while allowing greater diversification in accessory regions such as Antigen 1.

Finally, the observation that HUMC1 — a clinically associated hypervirulent isolate (23) — retains a full-length Zot that is structurally and partially sequence-conserved relative to ATCC 19606 strengthens the interpretation that Zot represents a biologically meaningful, functionally maintained virulence determinant rather than a degraded prophage remnant. The HUMC1–ATCC 19606 alignment therefore provides a mechanistic link between evolutionary conservation, domain-level structural constraint, and translational vaccine potential, positioning Zot as a promising candidate virulence-associated protein and immunological target, pending experimental validation—particularly its C-terminal Antigen 2 domain — as a “high-value” antigenic target within a rational reverse-vaccinology framework.

**Figure 4:**
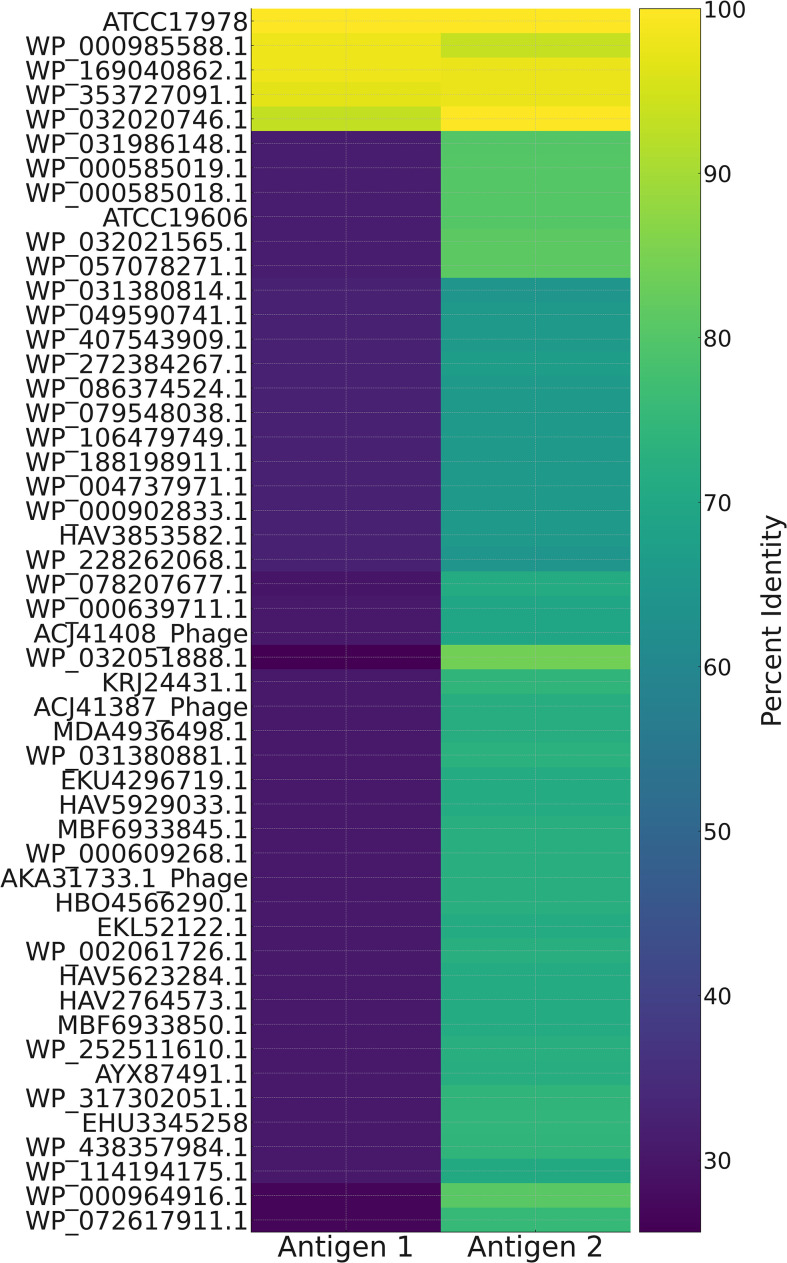
Heatmap of percent identity for Antigen 1 and Antigen 2 among *A. baumannii* strains and phage homologs, using HUMC1 as the anchor reveals Antigen to be highly conserved among 50 different strains. Each row represents a homologous sequence identified by BLASTp, and each column shows percent amino-acid identity to the HUMC1 antigen. Strains WP_032017345.1 and WP_009517212.1 lack Antigen 1 but retain Antigen 2. Conversely, strains CAA0160994.1_Phage, WP_000956750.1, WP_057689492.1, WP_098729704.1 WP_396233855.1, WP_169030959.1, WP_032017212.1, WP_231533305.1, and WP_072618039.1 encode Antigen 1 but lack Antigen 2. The asymmetric pattern highlights strong conservation of Antigen 2 across modern clinical and phage genomes, contrasted with the modular and variably present Antigen 1. The overall matrix demonstrates differential evolutionary stability between the two antigens and identifies strains where antigen loss or extreme divergence has occurred.

Comparative genomics reveals distinct conservation signatures of the two antigens across *A. baumannii* and associated phage. Comparative BLASTp analysis of Antigen 1 from *A. baumannii* HUMC1 strain, a ~101-amino-acid peptide, reveals a highly structured evolutionary distribution consistent with a conserved phage-derived domain disseminated across *A. baumannii* and associated phages. Using HUMC1 as the anchor, a total of 59 significant homologs were identified. At the apex of this distribution lies ATCC 17978, which exhibits 100% amino-acid identity and complete coverage of the 101-aa query, indicating that HUMC1 and ATCC 17978 encode an identical antigenic module. Four additional RefSeq proteins (WP accessions) form a closely related group with 93–98% identity and full-length alignments (**Figure 4**). The extremely high bit scores and E-values approaching 0 confirm that this “coreset” represents the canonical, contemporary antigen variant retained across modern nosocomial*A. baumannii* lineages (**Supplementary Table 1**).

Beyond this conserved core, the dataset shifts sharply toward a large cluster of hits (over 40 sequences) with ~30–33% identity and a smaller subset with ~36–38% identity (**Figure 4**). These proteins differ not in their homology signal—E-values remain strongly significant—but in their domain architecture. Many align only to a subregion of the HUMC1 antigen (typically residues 1–44), and their “coverage” values (~43–51%) indicate that the antigen-like region constitutes only half of a substantially larger protein, often 200+ amino acids in total length. This pattern reflects the classic signature of a phage-derived modular domain: a short, self-contained protein exists as an independent gene in some strains (e.g., HUMC1, ATCC 17978), but in many others the same domain is fused into larger multidomain proteins, likely through recombination or prophage genetic flux. The statistical robustness of these alignments (E-values ~10^-^¹¹ to 10^-14^) confirms that these are true distant homologs, not spurious partial matches (**Supplementary Table 1**) (103).

The ATCC 19606 homolog is particularly informative. Although the ATCC 19606 Zot protein is nearly twice the length of the HUMC1 Antigen 1 (~200 aa estimated), it aligns across 99 of the 101 HUMC1 residues, with an identity of ~31% and a bit score of ~55 (E ≈ 1.6 × 10^-14^) (**Supplementary Table 1**). This demonstrates a deep but unambiguous evolutionary relationship: ATCC 19606 retains a structurally recognizable Antigen 1–like domain but has accumulated extensive sequence divergence (~70% residue substitution) and has been incorporated into a larger protein context. Such divergence is characteristic of an ancestral, attenuated, or functionally remodeled variant, consistent with ATCC 19606’s status as an early, less virulent laboratory reference strain (23). Despite this divergence, the near-complete alignment length indicates conservation of the core tertiary scaffold, with variability concentrated in surface-exposed or non-essential residues (104, 105).

Phage-encoded sequences in the dataset reinforce this evolutionary model. Several phage proteins show ~30–38% identity to Antigen 1 and share the same partial-coverage pattern seen in divergent bacterial homologs. Their subject lengths (frequently ~200–225 aa, with a few shorter ~76-aa variants) again suggest that Antigen 1 represents a conserved structural module embedded within larger viral proteins. The presence of both bacterial and phage versions with similar identity profiles and domain boundaries indicates a continuous evolutionary bridge between prophage elements and chromosomal genes. The short, conserved alignments (qstart ~3–6 to qend ~44) observed in several phage and bacterial proteins point to a compact evolutionary core—a minimal motif that appears highly resistant to sequence erosion and likely represents the structural heart of the antigen.

Taken together, these observations position Antigen 1 as a phage-associated conserved domain family with distinct evolutionary tiers. In HUMC1 and ATCC 17978, Antigen 1 exists as a fully conserved, stand-alone protein corresponding to the modern canonical variant. In ATCC 19606 and numerous other strains, the same domain has undergone substantial divergence and has been integrated into larger polypeptides while retaining its essential fold. In phage genomes, Antigen 1 persists as part of even more complex architecture. The retention of high-confidence alignment across all tiers—despite identity levels ranging from 100% to as low as 30%—demonstrates a structurally indispensable core maintained across diverse genomic contexts (92, 106). From a biological standpoint, this architecture is characteristic of phage-derived effector or structural domains, which are frequently mobilized, fused, and repurposed through horizontal gene transfer (107–109). The HUMC1 Antigen 1 variant represents the most conserved extant form of this domain, whereas ATCC 19606 and phage-associated homologs define the evolutionary envelope of permissible divergence. This broader landscape provides a critical foundation for downstream analyses such as structural modeling, antigen prioritization, and reverse vaccinology.

Using HUMC1 as the query, the BLASTp hit table for Antigen 2 reveals a small, highly conserved protein family with strong sequence preservation across *A. baumannii* strains and several closely related phages. The query is a short protein of ~90 amino acids, and all 50 hits in the table represent statistically robust homologs (E-values spanning ~10^-69^ to 10^-^²^6^, bit scores ~84–191). Every hit aligns over at least ~76% of the HUMC1 sequence (qcovs ≥ 76.3%), with a median coverage of ~87% and many hits near or at full coverage (**Figure 4**; **Supplementary Table 2**). This immediately indicates that Antigen 2 corresponds to a compact, well-defined structural unit whose full length is largely conserved across the family, rather than a small motif embedded in very large, heterogeneous proteins.

At the top of the distribution sits the ATCC 17978 homolog, which is identical to HUMC1 Antigen 2 at the amino-acid level: it shows 100% identity over 90 aligned residues, with 0 mismatches, 0 gap openings, and 100% query coverage. Four additional sequences form a near-core cluster with 93–100% identity and full or nearly full coverage (length 88–90 aa, qcovs >95%) (**Figure 4**). The E-values for this group are on the order of 10^-69^-10^-64^, with bit scores between ~179 and 191, confirming that these sequences are not simply homologous but essentially represent the same protein in different strain backgrounds (**Supplementary Table 2**) (104). Together, HUMC1, ATCC 17978, and these closely related WP entries define a highly conserved canonical Antigen 2 variant that appears to be stably maintained in modern *A. baumannii* clinical lineages.

Below this inner core, most homologs fall in a high, but not identical, identity range. If we stratify the dataset, 4 hits are ≥95% identical, 1 lies between 90–95%, 8 between 80–90%, 23 between 70–80%, 14 between 60–70%, and only 2 fall just above the 50% threshold (53.9–54.8% identity) (**Figure 4**). Even these “lowest” identity hits remain well above the twilight zone of protein homology, and all align across substantial portions of the query (qcovs ~76–80%), indicating that every sequence is a confident homolog with a shared evolutionary origin (91) (**Supplementary Table 2**). Alignment lengths range from 70 to 91 residues, with an estimated subject length distribution centered around ~83–90 amino acids; most proteins in the family are thus roughly the same size as the query, differing only by small N- or C-terminal extensions or truncations. Importantly, qstart is overwhelmingly at positions 1–2 and qend at 87–90, showing that the alignment typically spans the entire length of both query and subject proteins with only modest terminal variation.

The ATCC 19606 homolog is particularly informative for understanding evolutionary distance within this Antigen 2 family. The ATCC 19606 sequence aligns to HUMC1 across 87 amino acids (qcovs ≈ 94.3%), with 80.46% identity, 17 mismatches, 0 gaps, and a bit score of 159 (E ≈ 5.4 × 10^-56^) (**Figure 4**; **Supplementary Table 2**). In contrast to Antigen 1—where the ATCC 19606 homolog dropped to ~31% identity—here Antigen 2 remains much more closely conserved between HUMC1 and ATCC 19606. The alignment is contiguous from residue 1 to 87 in both query and subject, with no insertions or deletions, implying that the overall length and architecture of the protein have been retained. At ~80% identity over nearly the full sequence, Antigen 2 in ATCC 19606 almost certainly shares the same three-dimensional fold and core functional properties as in HUMC1 and ATCC 17978. The differences that do exist are likely concentrated in surface-exposed residues, which may subtly modulate antigenicity or interaction surfaces without disrupting the structural scaffold.

Phage-encoded sequences further highlight the modular and mobile nature of Antigen 2. Three entries are explicitly annotated as phage proteins (e.g., ACJ41387_Phage, AKA31733.1_Phage, ACJ41408_Phage), all display high identity (≈69–72%) to HUMC1 Antigen 2, with alignment lengths of 87–91 aa and coverage values around 86–87% (**Figure 4**; **Supplementary Table 2**). Their subject-length estimates (~87–89 aa) are essentially indistinguishable from those of the bacterial homologs, indicating that, unlike Antigen 1, Antigen 2 generally does not appear as a small domain fused into a much larger polypeptide. Instead, it is preserved as a near full-length stand-alone module in both bacterial and phage genomes. The fact that phage and bacterial variants share such strong similarity and almost identical sizes argues that Antigen 2 represents a compact phage-derived protein that has been transferred into *A. baumannii* with minimal structural remodeling (108, 109). In evolutionary terms, these phage homologs are not distant relics but actively conserved members of the same protein family, suggesting ongoing or relatively recent phage–bacterium gene exchange (107, 110).

Overall, the bit score and identity landscape for Antigen 2 paints a picture of a tightly conserved, short protein family with relatively limited diversification. All 50 hits are robust homologs; none fall into the highly degenerate range typical of ancient or partially decayed genes. The variation that does exist—identity dropping from 100% to ~54%—occurs against a backdrop of consistently high coverage and similar protein lengths, which is exactly what one would expect for a functionally important small protein under moderate-to-strong purifying selection (92, 111). HUMC1 and ATCC 17978 occupy the center of this family, encoding an identical canonical variant; ATCC 19606 and other *A. baumannii* isolates maintain closely related versions (~70–85% identity); and several phages contribute parallel homologs with comparable sequence conservation. This pattern strongly suggests that Antigen 2 corresponds to a structurally rigid, functionally constrained phage-associated protein that has been horizontally disseminated but not extensively remodeled.

It should be noted that a subset of strains in the BLASTp dataset lacked detectable homologs for either Antigen 1 or Antigen 2. This absence reflects true evolutionary patterns rather than technical errors (**Figure 4**; **Supplementary Table 1** and **S2**) (112, 113). The contrasting presence/absence patterns highlight the different evolutionary histories of these two antigens: Antigen 1 is highly variable, often fused into larger proteins or embedded within decayed phage elements, and is therefore more frequently lost or undetectable (114). In contrast, Antigen 2 represents a compact, structurally conserved phage-derived module that is typically retained across *A. baumannii* and phage genomes, with only a few divergent strains lacking it (114). Together, these patterns reveal a mosaic distribution shaped by phage-mediated mobility, domain fusion, and selective retention, providing important context for antigen conservation and reverse-vaccinology design.

From a reverse-vaccinology perspective, these evolutionary features are particularly compelling. Vaccine antigens that are short, structurally compact, and conserved across genetically diverse strains are less likely to undergo immune-driven escape and more likely to elicit cross-strain immune recognition (115). Antigen 2 satisfies each of these criteria: it is consistently present as a ~90-aa protein across the dataset, retains high identity even between historically and clinically distinct isolates, and shows little evidence of truncation, fusion, or domain reshuffling. These observations collectively suggest that Antigen 2 may perform a conserved biological function that constrains its structural tolerance for variation (8, 36, 90) — precisely the evolutionary context in which stable B- and T-cell epitopes are most likely to be maintained.

Thus, Antigen 2 does not merely appear conserved — it displays the hallmarks of a rational vaccine candidate: genomic retention across strains, limited structural plasticity, and a high barrier to sequence disruption. When integrated with immunoinformatic epitope-prediction pipelines and downstream structural modeling, these alignment data provide a strong basis for prioritizing Antigen 2 as a high-confidence, broadly conserved antigenic target within *A. baumannii* reverse-vaccinology frameworks.

**Figure 5:**
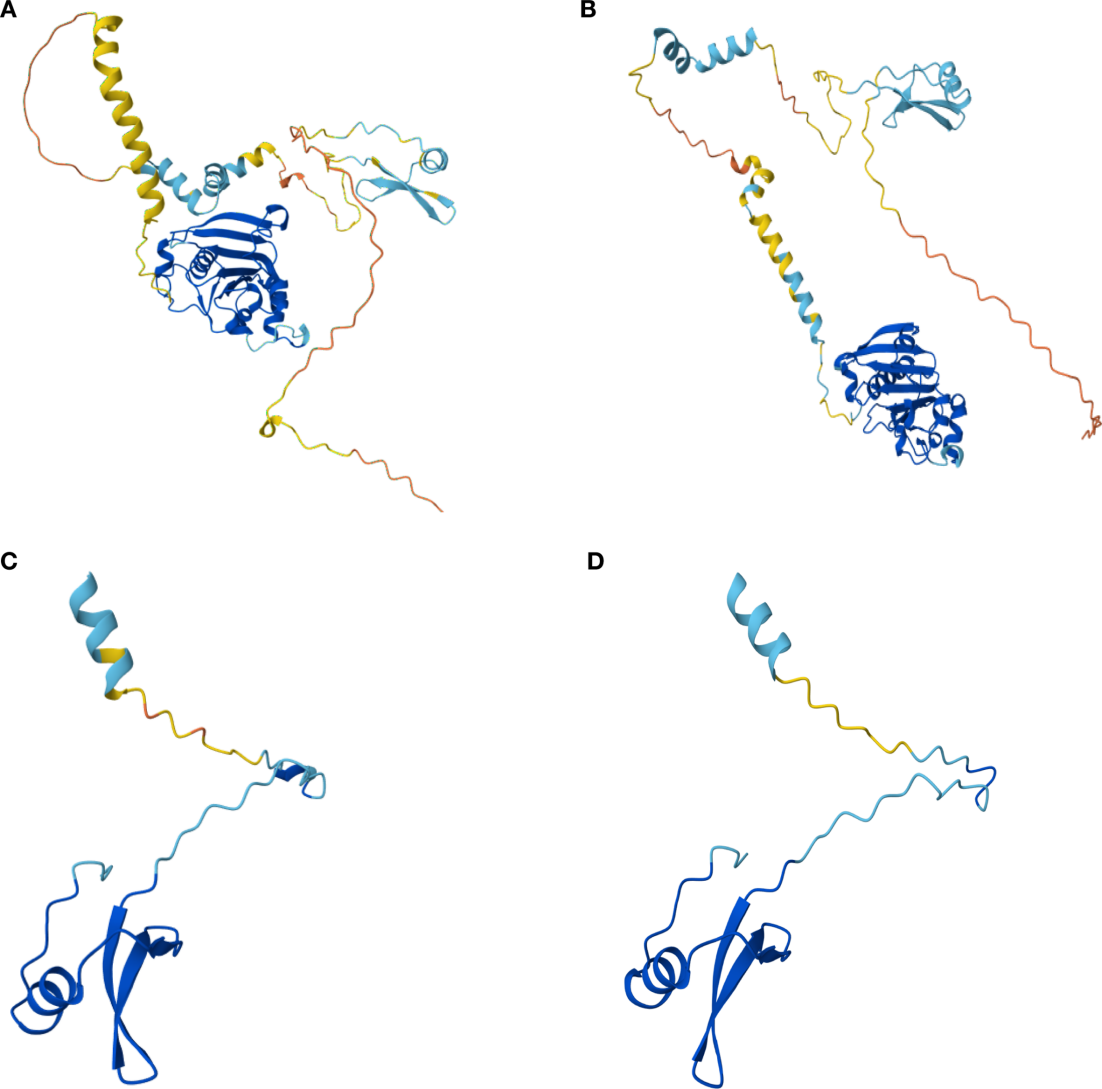
AlphaFold3-predicted structural models of full-length Zot and C-terminal Antigen 2 demonstrate conserved architecture across divergent *A. baumannii* strains. Structural models of full-length Zot from *A. baumannii* HUMC1 **(A)** and ATCC 19606 **(B)**, generated using AlphaFold3, reveal highly similar tertiary architectures despite substantial primary-sequence divergence (~38–40% identity). Backbone organization, including β-sheet assemblies and associated helical regions, is preserved across both strains, indicating conservation of the global fold. Predicted Local Distance Difference Test (pLDDT) confidence scores are mapped onto the structures, with warmer colors indicating higher confidence. The C-terminal region exhibits consistently high confidence (pLDDT ≥80–90), whereas portions of the N-terminal region display comparatively lower confidence, suggesting increased conformational flexibility. Structural models of the isolated C-terminal Antigen 2 domain from HUMC1 **(C)** and ATCC 19606 **(D)** demonstrate a compact and structurally cohesive architecture that is highly conserved across strains. Predicted Aligned Error (PAE) patterns (not shown) further support low structural uncertainty within this domain relative to more variable regions of the full-length protein. The strong agreement between sequence conservation and structural stability in Antigen 2 supports its prioritization as a candidate region for downstream experimental evaluation. All structures represent AlphaFold3-predicted conformations and should be interpreted as high-confidence structural models rather than experimentally determined structures.

AlphaFold3-based structural analysis reveals conserved architecture and epitope localization in Zot. To assess whether sequence divergence impacts structural integrity, high-confidence models of full-length Zot and the C-terminal Antigen 2 domain were generated using the deep learning–based framework AlphaFold3 (**Figure 5**) (116). Representative sequences from the HUMC1 and ATCC 19606 strains—sharing ~38–40% amino acid identity—were selected to evaluate structural conservation across evolutionarily distant lineages. Despite substantial primary-sequence divergence, the predicted structures exhibited a highly similar overall tertiary architecture. Backbone superposition revealed preservation of key secondary structural elements, including β-sheet organization and associated helical regions, indicating that the global fold is maintained across strains. This pattern is consistent with the presence of an evolutionarily conserved structural scaffold underlying sequence variability (**Figures 5A, B**).

Model confidence metrics further supported this interpretation. Predicted Local Distance Difference Test (pLDDT) scores were consistently high across the C-terminal domain (Antigen 2), frequently exceeding 80–90, indicating strong confidence in local structural accuracy (**Figures 5C, D**). In contrast, portions of the N-terminal region displayed moderately reduced confidence, suggesting greater conformational flexibility or reduced structural constraint. Predicted Aligned Error (PAE) analysis reinforced this domain-level distinction, with low inter-residue error observed within the C-terminal domain and comparatively higher uncertainty in more variable regions. Domain-specific modeling of Antigen 2 revealed a compact and structurally cohesive architecture that remained highly consistent across strains, supporting sequence-based observations of elevated conservation and indicating stronger structural constraint relative to the N-terminal region.

To integrate structural and immunological features, predicted B-cell and T-cell epitopes were mapped onto the AlphaFold3-derived models. Predicted epitope regions frequently localized to solvent-accessible surfaces and loop-associated regions, consistent with features associated with immune recognition. Several high-confidence T-cell epitopes identified within conserved regions of Antigen 2 corresponded to structurally well-defined regions with high pLDDT scores, suggesting that these motifs are embedded within stable structural elements. In contrast, certain predicted B-cell epitope clusters aligned with regions of moderate confidence and elevated PAE, potentially reflecting increased flexibility, a feature often associated with antibody-accessible conformations.

While AlphaFold3 provides high-accuracy structural predictions, these models represent static conformations and do not capture full protein dynamics or environmental influences (116, 117). Accordingly, the observed structural conservation and epitope localization should be interpreted as probabilistic and hypothesis-generating rather than definitive. Nevertheless, the convergence of sequence conservation, structural stability, and predicted epitope distribution provides a coherent framework for prioritizing specific regions of Zot—particularly within the C-terminal Antigen 2 domain—for downstream experimental validation.

**Figure 6:**
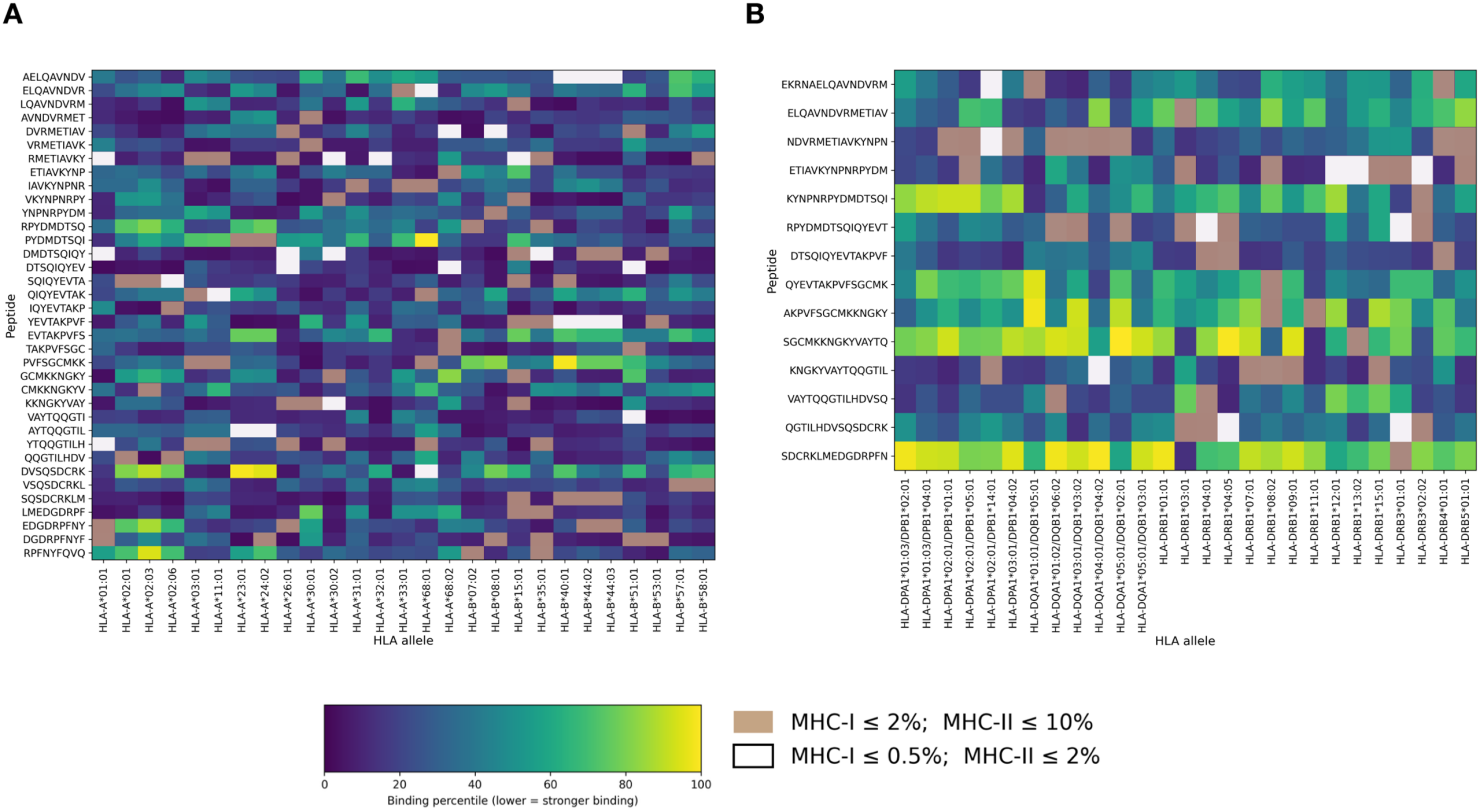
Predicted peptide–HLA binding landscape for candidate *A. baumannii* antigens across MHC Class I and Class II alleles. Heatmaps display the predicted binding behavior of all antigen-derived peptides that demonstrated at least one positive HLA interaction across a representative panel of human Class I **(A)** and Class II **(B)** alleles. The viridis background gradient (purple → green → yellow) denotes the continuous NetMHCpan/NetMHCIIpan binding percentile score for each peptide–allele pair, thereby illustrating the full quantitative affinity spectrum independent of thresholding. Lower percentile scores correspond to stronger predicted peptide–HLA interactions. Threshold-defined binders are overlaid onto the background as uniform color blocks. Light-brown shading identifies standard-affinity binders (MHC-I ≤2%; MHC-II ≤10%), representing peptides predicted to be reliably processed and presented. White shading marks high-affinity binders (MHC-I ≤0.5%; MHC-II ≤2%), corresponding to epitopes with the strongest predicted anchor compatibility and highest likelihood of immunodominance. Only peptides with at least one predicted binding interaction are shown; rows corresponding to non-binding peptides were removed for clarity. Vertical or horizontal clusters of white and light-brown overlays indicate regions enriched for multi-allelic binding, consistent with conserved epitope cores capable of engaging diverse HLA repertoires. Peptides demonstrating both MHC Class I and Class II binding highlight antigen segments with dual-pathway presentation potential, supporting coordinated induction of CD8^+^ and CD4^+^ T-cell responses. Together, these data identify discrete antigen regions containing densely distributed high-confidence epitopes appropriate for downstream prioritization in reverse-vaccinology pipelines.

Antigen 2 contains highly conserved high-affinity MHC-I and MHC-I epitopes. A high-resolution, allele-resolved mapping of peptide–MHC interactions was performed across both Class I and Class II MHC molecules to dissect the intrinsic immunogenic architecture of Antigen 2. The viridis background (representing continuous percentile-based binding energies derived from NetMHCpan and NetMHCIIpan algorithms) revealed the full thermodynamic spectrum of predicted peptide–HLA interactions, enabling differentiation between general binding propensities and threshold-defined biologically actionable epitopes (**Figure 6**). This un-thresholded landscape demonstrated that peptide–HLA engagement is highly heterogeneous and structurally constrained, with distinct distributional signatures for MHC-I versus MHC-II pathways.

The MHC Class I binding matrix exhibited the classical hallmarks of MHC-I-restricted antigen presentation: narrow registers of permissive nonameric cores, strong allele-specificity, and punctate distribution of high-affinity anchor residues (118). The viridis background reflected predominantly high percentile values (indicating weaker predicted binding), consistent with strict constraints imposed by the closed MHC-I binding groove and the reliance on canonical anchor residues at P2 and PΩ (100, 102). Despite this, several peptides demonstrated substantial reductions in predicted percentile scores across multiple HLA-A, -B, and -C alleles, suggesting the presence of conserved hydrophobic anchor motifs compatible with broad MHC Class I presentation (119, 120).

Threshold overlays further refined this structural topography. Beige-marked ≤2% binders were widely distributed across the allele panel, indicating multiple peptides with nonameric motifs capable of engaging diverse MHC-I pockets (**Figure 6A**) (121). More importantly, a subset of epitopes crossed the stringent ≤0.5% affinity threshold (represented in white), yielding discrete, high-affinity interaction nodes (121). These high-affinity zones often colocalized with regions of elevated viridis intensity, suggesting congruence between predicted structural accommodation and quantitative energetic favorability (122). Several of these epitopes demonstrated cross-supertype binding (e.g., HLA-A*02, A*03, B*07 clusters), a property strongly associated with immunodominance and enhanced population-level coverage (119, 120, 123).

Consistent with the biophysical properties of the MHC-I groove, the Class II binding map revealed a markedly more diffuse affinity distribution with extensive regions of intermediate-to-strong viridis intensities (**Figure 6B**). The open-ended nature of the HLA-DP, -DQ, and -DR binding pockets allows for multiple overlapping 13–17 aa registers to be accommodated, producing hallmark patterns of epitope promiscuity (124). Beige threshold overlays (≤10% binders) were prevalent across nearly all DRB1, DQA1/DQB1, and DPA1/DPB1 alleles examined, indicating that the antigen contains a substantial repertoire of CD4^+^ T-cell epitopes capable of presentation across genetically diverse human populations. White high-affinity cores (≤2%) appeared as concentrated foci, representing register-stabilizing motifs predicted to engage the P1, P4, P6, and P9 anchor pockets of MHC-II with high energetic favorability (102). These epitopes frequently aligned with contaminant-free conserved sequence motifs within the antigen, implying selective pressure for structural stability and immunogenic retention. The formation of these high-affinity MHC-II hotspots is strongly associated with efficient CD4^+^ T cell priming, cytokine polarization, and enhancement of downstream B-cell responses through T follicular helper (Tfh) cell interactions (125).

A key observation was the identification of several peptides exhibiting simultaneous MHC-I and MHC-II binding signatures, including overlapping white and beige zones across both heatmaps (**Figure 6**). These dual-reactive epitopes are of immunological interest because they support synchronous activation of cytotoxic CD8^+^ T cells and helper CD4^+^ T cells. Such coordination is essential for sustained CTL expansion, memory establishment, and robust antibody class switching (126). The convergence of binding across both antigen presentation pathways suggests that certain regions of the antigen contain evolutionarily conserved amino acid motifs capable of assuming multiple structural registers—a criterion often associated with immunodominant epitope clusters.

Altogether, the combined heatmaps reveal a sophisticated immunogenic architecture characterized by stringent but strategically positioned MHC-I high-affinity pockets, widespread and promiscuous MHC-II recognition domains, and several dual-class epitope regions with high translational relevance. The presence of cross-allelic high-affinity binders and overlapping epitope clusters support the prioritization of Antigen 2 for further investigation in multi-epitope vaccine design, particularly for constructs aiming to elicit coordinated CD4^+^ and CD8^+^ responses.

High-stringency MHC-I and MHC-II filtering reveals a restricted but immunologically potent set of shared epitope cores. To delineate the antigenic regions most likely to elicit robust and broadly distributed T-cell immunity, we applied a high-stringency epitope prediction workflow integrating both MHC Class I and Class II binding models. We focused the analysis on only the strongest and most biologically meaningful binders. This stringent approach sharply narrowed the full epitope landscape to a refined set of high-confidence peptide motifs. Importantly, several of these motifs overlapped between MHC-I and MHC-II pathways, indicating the presence of conserved regions that are simultaneously accessible to both cytosolic and endosomal antigen-processing routes. The subsequent shared-core heatmap analysis provides a detailed visualization of these dual-class epitopes, highlighting their distribution, allelic coverage, and relative binding strengths across a diverse panel of HLA molecules.

**Figure 7:**
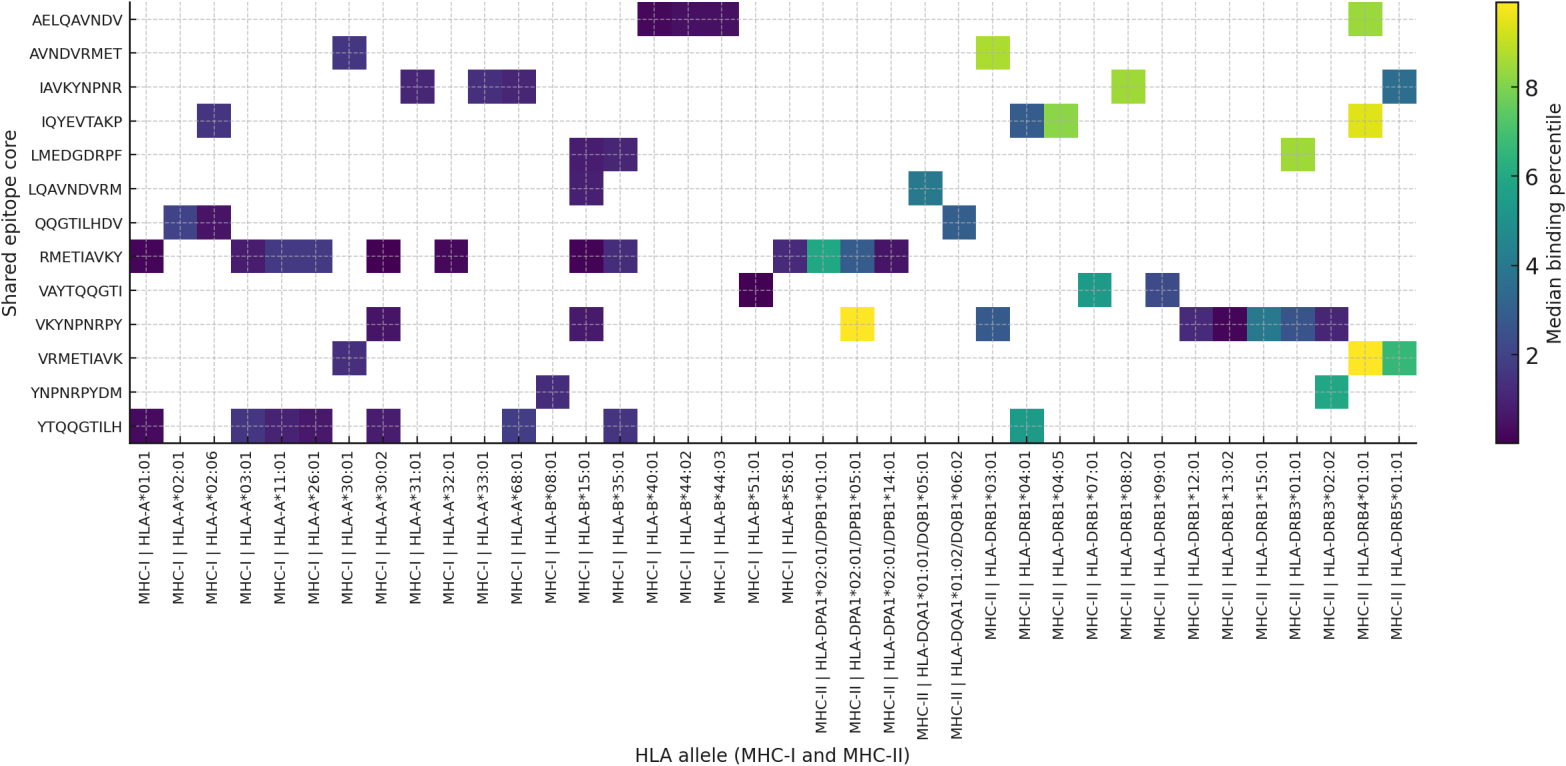
Shared-core epitope binding heatmap generated using high-stringency MHC-I and MHC-II prediction thresholds demonstrate the presence of 13 overlapping epitopes. Heatmap displaying the binding intensities of all epitope cores simultaneously predicted to bind both MHC Class I (filtered at ≤2% median binding percentile) and MHC Class II (filtered at ≤10%). Only peptide cores present in both filtered datasets were retained, resulting in a refined set of 13 shared epitopes. Columns represent individual HLA alleles from both classes, with “MHC-I | allele” and “MHC-II | allele” prefixes indicating class identity. Rows correspond to shared epitope cores. Colors reflect the predicted binding percentiles on a viridis color scale, where dark purple/blue denotes the strongest predicted binders (lowest percentile ranks) and green to yellow represents comparatively weaker binders within the filtered high-affinity range. The heatmap highlights conserved antigenic regions capable of being processed through both cytosolic (MHC-I) and endosomal (MHC-II) pathways and demonstrates broad HLA allelic coverage across multiple families. These dual-class, high-confidence epitopes represent prime predicted immunodominant motifs for multi-epitope vaccine design.

High-stringency epitope prediction identified a remarkably conserved and immunologically significant subset of 13 shared epitope cores that simultaneously passed the strict dual thresholds of ≤2% binding percentile for MHC-I and ≤10% for MHC-II (**Figure 7**). These epitopes represent the intersection where the antigen is efficiently processed and presented through both MHC Class I and Class II, thereby maximizing the probability of coordinated CD8^+^ and CD4^+^ T-cell activation. Importantly, these shared cores spanned all major functional and structural regions of the antigen, including the N-terminal signal-associated region, the central NP–RPY and YEVTAKPVF-containing loops, and the conserved C-terminal domain. Together, these 13 epitopes demonstrated binding coverage across 38 distinct HLA alleles, encompassing a wide range of A-, B-, DR-, DQ, and DP-locus haplotypes, supporting their relevance for broad population-level vaccine coverage.

Several epitopes stood out for their exceptionally strong and balanced presentation across both classes. RMETIAVKY, VAYTQQGTI, and VKYNPNRPY, located within the highly conserved central region of Antigen 2, exhibited some of the lowest combined binding percentile values across the entire dataset. For these cores, MHC-I binding percentiles frequently clustered between 0.01–0.20%, demonstrating ultra-high predicted affinity across alleles such as HLA-A*01:01, A*30:02, A*68:01, B*15:01, and B*40:01.* Simultaneously, these same cores achieved MHC-II binding percentiles in the 0.14–2.0% range for DRB1*13:02*, DRB112:01, DRB301:01, DQA101:01/DQB105:01, and DPA1/DPB1 haplotypes, positioning them among the strongest dual-class binders within the antigen (**Figure 7**). Their capacity to engage both MHC classes at high affinity suggests that these epitopes may function as immunodominant T-cell activation clusters, driving the initiation of both cytotoxic and helper responses through coordinated antigen presentation.

Another subset of shared cores—IQYEVTAKP, YEVTAKPVF, and IAVKYNPNR—demonstrated broader but moderately less intense MHC-II binding (typically 3.0–9.8%), while still maintaining robust MHC-I affinity (≤0.5–1.0%) (**Figure 7**). These epitopes displayed binding across 6–10 alleles, reflecting widespread compatibility even though their MHC Class II scores were not as low as those of the highest-ranked epitopes. Their presence in both classes confirms that even moderately strong MHC-II binders can be highly valuable when anchored by strong MHC-I presentation, as such epitopes are known to promote more durable and polyfunctional T-cell responses due to their simultaneous engagement of CD4^+^ and CD8^+^ compartments.

In addition, several shared epitopes within the N-terminal and C-terminal regions of Antigen 2—such as AELQAVNDV, LQAVNDVRM, AVNDVRMET, and LMEDGDRPF—exhibited balanced dual-class presentation despite somewhat narrower allele breadth (typically 3–6 alleles). For these epitopes, MHC-I percentile values generally fell between 0.3–1.5% while MHC-II binding ranged from 4–9%, indicating reliable, though less promiscuous, antigen presentation (**Figure 7**). The fact that these regions still passed the stringent dual-class criteria indicates that they contribute meaningful redundancy to the epitope architecture—an important characteristic for preventing immune escape in the face of sequence variation. Notably, several of these epitopes overlap with highly conserved stretches of the antigen that demonstrate minimal sequence variability across strains, making them particularly attractive components for a universal or broad-spectrum vaccine design.

When each of the 13 shared cores was examined individually, the total number of HLA alleles bound per epitope ranged from 3 to 15, with an average of 7–9 alleles per core. This degree of coverage is unusually high for a bacterial antigen and indicates that the antigen contains multiple independent “anchor points” for T-cell recognition. Importantly, the shared-core network is not confined to a single region but instead distributed across the Antigen 2, forming a highly redundant immunogenic architecture. Such architecture is characteristic of successful subunit vaccine targets, as it provides multiple, strain-conserved epitopes capable of generating CD8^+^ cytotoxicity, CD4^+^ helper activation, long-lasting memory formation, and robust antigen boosting.

Altogether, the convergence of strong MHC-I and MHC-II binding onto the same 13 conserved cores provides compelling evidence that the antigen possesses an unusually dense and overlapping T-cell epitope structure. These shared cores represent the most immunologically potent and vaccine-relevant elements of the antigen, with the ability to activate both major T-cell arms across genetically diverse human populations. Their combined high affinity, multi-allelic breadth, and structural conservation indicate that they form the backbone of an effective, cross-protective, and predicted to support T-cell–driven immune response, thereby supporting Antigen 2 as a high-priority candidate for experimental validation.

Based on the integration of sequence conservation, structural stability, and high-affinity MHC binding, we propose a rational multi-epitope vaccine design centered on the Antigen 2 domain. High-confidence shared epitopes demonstrating dual MHC Class I and II binding were prioritized to maximize coordinated CD8^+^ and CD4^+^ T-cell responses. The selected epitope set provides broad HLA allele coverage and is enriched in structurally constrained regions, minimizing the likelihood of immune escape. This framework supports the development of a compact, conserved, and immunologically robust vaccine construct targeting diverse *A. baumannii* strains.

**Figure 8:**
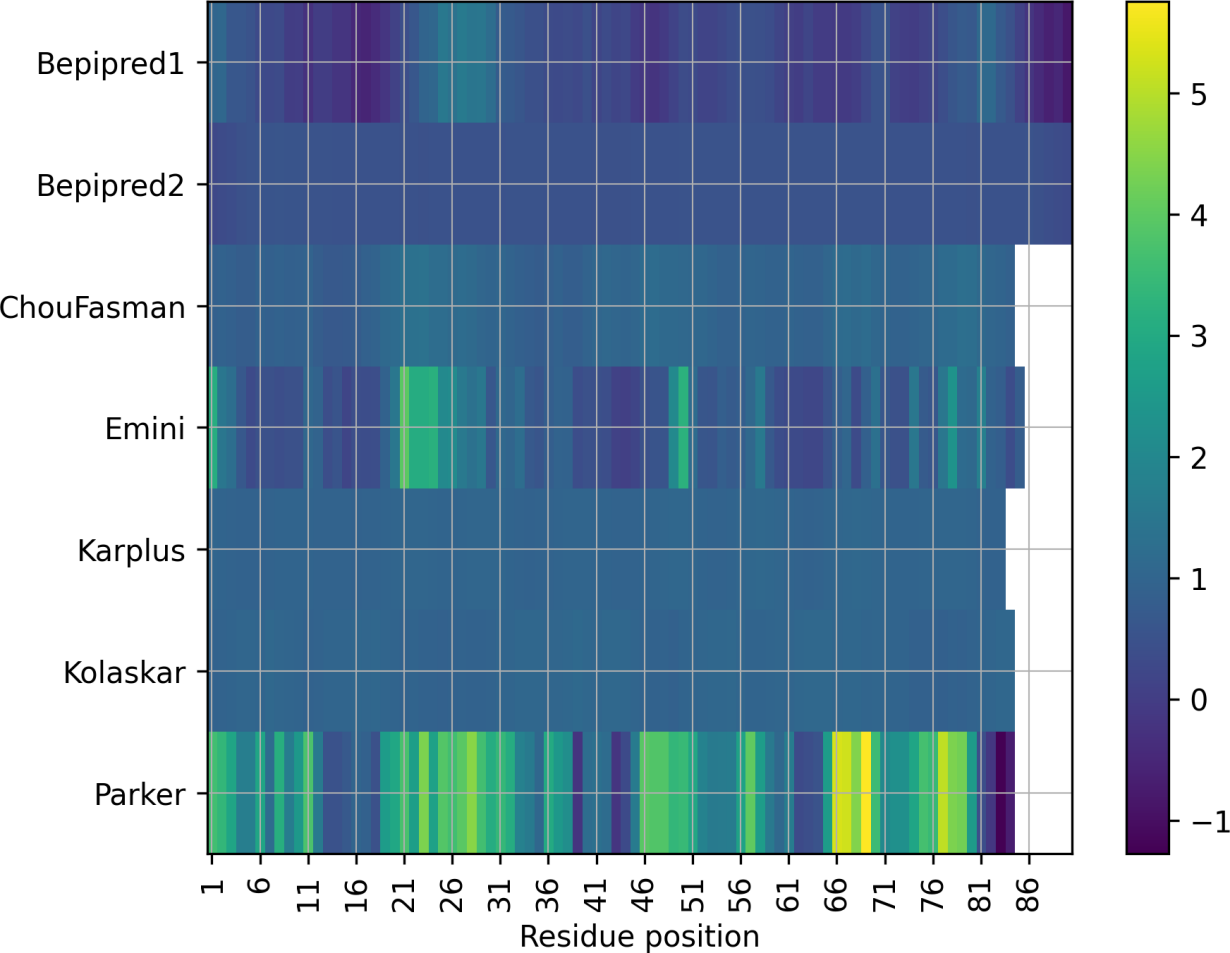
Integrated B-cell epitope prediction heatmap (raw scores). Heatmap showing the distribution of predicted linear B-cell epitopes across the protein sequence based on seven independent algorithms. Rows correspond to Bepipred 1.0, Bepipred 2.0, Chou–Fasman β-turn propensity, Emini surface accessibility, Karplus–Schulz flexibility, Kolaskar–Tongaonkar antigenicity, and Parker hydrophilicity. Columns represent residue position along the antigen. The color legend shows epitope-favorability strength. Color intensity reflects the raw per-residue prediction score returned by each algorithm, with warmer colors indicating higher predicted epitope probability or more favorable physicochemical characteristics for antibody recognition. Cooler colors indicate weaker or unfavorable signal. Gridlines and down-sampled x-axis labels are included to facilitate alignment of local peaks across algorithms. Several discrete multi-algorithm “hotspot” regions are visible, indicating spatial clustering of residues that score highly across distinct predictive frameworks, whereas other regions display uniformly low values across all tools. Together, the heatmap provides a consensus-level view of where B-cell epitope–like features concentrate along the sequence prior to normalization or thresholding.

Quantitative integration of B-cell epitope features in a reverse vaccinology framework. To prioritize linear B-cell epitopes within a reverse vaccinology framework, we generated an integrated epitope landscape by combining per-residue predictions from seven independent algorithms (**Figure 8**). These included Bepipred 1.0 and Bepipred 2.0 (127), which infer antigenicity using empirical and machine-learning models, together with tools that quantify intrinsic structural favorability for antibody recognition: Chou–Fasman β-turn propensity (128), Emini Surface Accessibility (129), Karplus–Schulz Flexibility (130), Kolaskar–Tongaonkar Antigenicity (131), and Parker Hydrophilicity (132). The resulting values were aligned across the sequence and visualized as a heatmap in which rows represent prediction algorithms and the x-axis corresponds to amino-acid position (**Figure 8**). Higher color intensity reflects greater predicted epitope likelihood or favorable physicochemical properties.

Across the protein, epitope-associated features were not evenly distributed, but instead clustered into discrete high-signal regions. Quantitatively, Bepipred 1.0 scores spanned approximately −0.8 to +1.3, with most residues centered near 0, whereas putative epitope residues consistently exceeded ~0.5–0.7. Bepipred 2.0 epitope probability values ranged from ~0.25–0.65, with residues predicted to be immunogenic typically ≥0.50. When these values were visualized together with hydrophilicity, flexibility, and accessibility signals, three major consensus epitope clusters were observed (**Figure 8**).

The first and strongest cluster was located between positions ~10–25. In this region, Bepipred 1.0 scores frequently reached 0.8–1.0, and Bepipred 2.0 probabilities were elevated to 0.50–0.60. These residues also demonstrated markedly increased solvent accessibility (Emini index ~1.2–3.1; baseline = 1.0) and strong hydrophilicity (Parker index ~2.5–3.6). Local structural permissiveness was supported by flexibility values of ~1.00–1.05 compared with a background mean of ~0.98–0.99, along with β-turn propensity values approaching ≥0.85–0.90. The second major hotspot was located between positions ~35–50. Here, Bepipred 1.0 values were typically ≥0.6–0.9 and Bepipred 2.0 reached ~0.48–0.55, coinciding with elevated Emini (~1.3–2.2) and Parker (~1.5–2.8) values. Notably, Kolaskar–Tongaonkar antigenicity frequently exceeded the classical threshold of 1.00 in this region (observed range ~1.03–1.08), further supporting likely antigenicity. A third, moderately strong cluster appeared between positions ~65–80, where Bepipred 1.0 values remained mostly >0.4 (with local peaks near 0.8) and Bepipred 2.0 values fluctuated around 0.45–0.55; these residues again demonstrated elevated hydrophilicity (~1.7–2.6) and accessibility (~1.2–1.9) compared with surrounding regions.

In contrast, regions outside these clusters consistently displayed weak predictive support across nearly all algorithms. These positions were typically characterized by Bepipred 1.0 scores <0.2, Bepipred 2.0 values ~0.30–0.40, Emini accessibility ≤1.0, Parker hydrophilicity ≤1.0–1.5, and Karplus–Schulz flexibility values <0.98. The convergence of low scores across independent predictors suggests structurally rigid, buried, or hydrophobic regions that are less likely to contribute substantially to antibody recognition.

Within the reverse vaccinology framework, the most informative metric is not the performance of any single algorithm, but the agreement across orthogonal, biologically distinct predictors (133). The three highlighted regions satisfy this requirement: they are predicted to be antigenic by both Bepipred models, solvent-exposed and hydrophilic, structurally flexible, enriched in β-turn character, and above empirical antigenicity thresholds. This convergence reduces reliance on any single predictor and highlights regions consistently supported across multiple biophysical features and defines a consensus-derived positive design space composed of residues most likely to function as linear B-cell epitopes (134).

Thus, the integrated heatmap acts as a quantitative immuno-biophysical filter, condensing the full protein sequence into a small number of rational, high-confidence target regions. These consensus clusters represent the most promising candidates for downstream vaccine design, including peptide synthesis, structural epitope mapping, multiepitope construct assembly, and experimental validation in serological assays.

**Figure 9:**
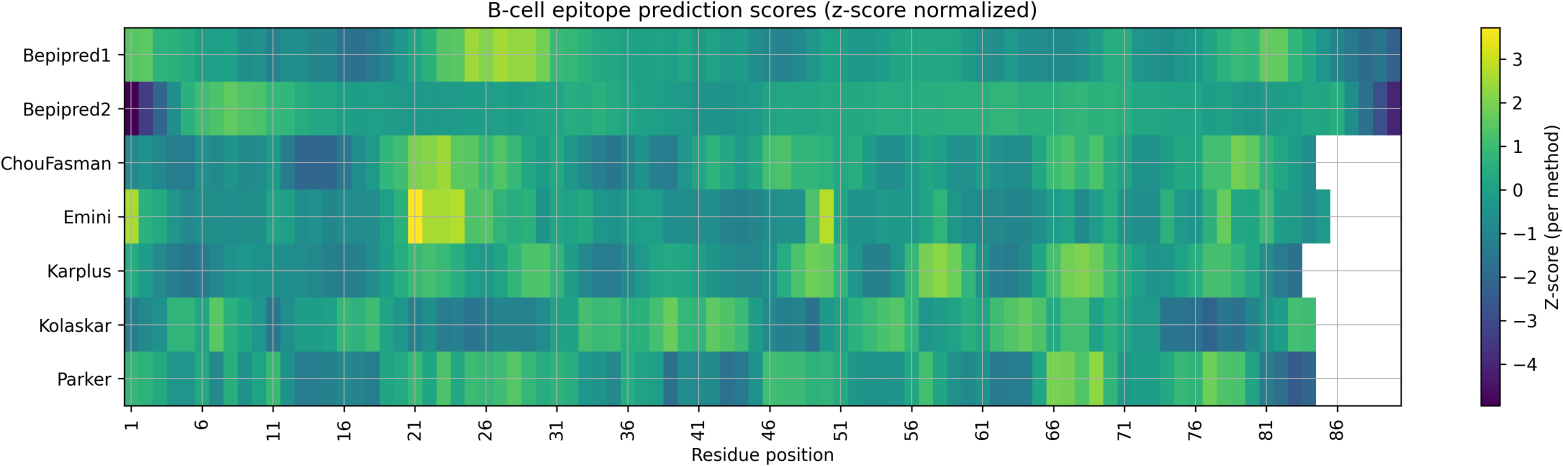
z-score–normalized heatmap prediction profiles standardized across the protein sequence. Because the seven prediction algorithms use distinct numerical scoring systems, a normalized heatmap was also generated to enable direct comparison of relative epitope signal across methods. For each algorithm independently, the raw per−residue scores were converted to z−scores across the protein sequence using the transformation z = (x − mean)/SD, where x is the residue−level score, mean is the dataset mean for that algorithm, and SD is the corresponding standard deviation. This standardization centers each method around zero and expresses each position in units of standard deviation relative to that method’s mean. Positive z−scores therefore indicate residues that score above the algorithm−specific average, whereas negative values indicate below−average epitope−associated signal. The normalized heatmap was constructed using the same visualization parameters as the raw heatmap, but the color legend represents standardized rather than absolute scores. This approach places all algorithms onto a common relative scale and improves the contrast between consensus epitope hotspots and background regions.

To enable comparison of relative epitope trends across algorithms with different numerical scales, we generated a z-score–normalized heatmap in which each prediction profile was standardized across the protein sequence (**Figure 9**). Normalization revealed that all seven algorithms converged on the same discrete epitope-enriched regions, but with clearer contrast between locally high- and low-scoring segments. Three dominant consensus clusters were observed, centered approximately at positions ~10–25, ~35–50, and ~65–80, where normalized values were consistently positive (z ≥ 0.5–1.5) across ≥4–6 predictors. These regions therefore represent sites where residues performed substantially above the method-specific mean, independent of absolute score range. In contrast, large portions of the remaining sequence displayed uniformly negative normalized values (z ≤ −0.5), indicating method-wide depletion of epitope-favorable properties. Thus, normalization sharpened the discrimination between high-confidence epitope “hotspots” and background regions, further reinforcing that epitope-like features are spatially concentrated rather than evenly distributed along the antigen. For the normalized heatmap, scores from each algorithm were converted to z-scores across the protein sequence, such that warmer colors indicate residues that scored above the method-specific mean and cooler colors indicate below-average scores, enabling cross-method comparison on a common scale.” Because several of the underlying predictors capture related physicochemical properties, the consensus signal should be interpreted as convergence across complementary descriptors rather than independent statistical evidence.

## Discussion

The emergence of MDR *A. baumannii* as a leading cause of nosocomial infections has intensified the need for alternative therapeutic strategies that function independently of antibiotic susceptibility (135, 136). In this study, we applied an integrated immunoinformatic and evolutionary framework to evaluate the Zot as a candidate for immune-based targeting. Importantly, it should be noted that while Zot has been extensively characterized as a virulence factor in *V. cholerae*, its role in *A. baumannii* remains incompletely defined. The presence of Zot homologs in *A. baumannii* and other GNB pathogens has been recently proposed, including by our group (23, 24), but direct experimental validation of its contribution to virulence in *A. baumannii* is currently under investigation. Therefore, the interpretations presented here should be viewed within the context of a hypothesis-generating framework, in which Zot represents a candidate virulence-associated protein warranting further functional assessment. Our findings demonstrate that Zot combines structural conservation, domain-specific evolutionary constraint, and a dense immunogenic architecture, supporting its prioritization as a candidate for experimental evaluation as a multi-epitope vaccine target.

A central finding of this study is the presence of structure-based constraint acting on Zot despite substantial primary sequence divergence between strains. Comparative analyses demonstrated that divergent homologs retain near complete alignment continuity and conserved tertiary structure, indicating preservation of shared functional fold. This pattern is characteristic of proteins under strong structural constraint, where functional requirements limit allowable sequence variation. Importantly, this constraint is not uniform: the C-terminal domain (Antigen 2) exhibits markedly higher conservation and structural stability compared to the N-terminal region.

From an immunological perspective, the most significant finding is the identification of a highly organized and overlapping epitope architecture within Antigen 2. High-resolution MHC binding analysis revealed a concentrated network of high-affinity MHC Class I and Class II epitopes, including 13 shared epitope cores that meet stringent dual-binding criteria. These epitopes demonstrate broad HLA allele coverage and are distributed across conserved structural regions, suggesting a high likelihood of population-level immunogenicity. The presence of dual MHC Class I and II binding motifs is particularly important, as it enables coordinated activation of CD8^+^ cytotoxic T cells and CD4^+^ helper T cells—an essential feature for effective and durable adaptive immune responses (137). Such dual-pathway engagement is strongly associated with enhanced memory formation, improved antibody class switching, and sustained effector function (138).

Notably, several of the highest-affinity shared epitopes are localized within structurally constrained regions of the protein, reinforcing the link between evolutionary conservation and immunogenic potential (139, 140). This convergence suggests that key functional regions of Zot are simultaneously preserved for structural integrity and exposed to immune recognition, creating an optimal landscape for vaccine targeting. In contrast to many bacterial antigens that exhibit high variability or immune-driven escape, Zot—particularly within its C-terminal domain—appears to maintain a relatively high barrier to sequence disruption. This property significantly enhances its suitability as a cross-protective antigen capable of eliciting immune responses across genetically diverse *A. baumannii* strains.

Integration of B-cell epitope prediction further supports this immunological profile. Consensus-based analysis across multiple predictive algorithms identified discrete regions enriched in surface accessibility, hydrophilicity, flexibility, and antigenicity—hallmarks of effective antibody targets (127–132). Although overlap between B-cell and T-cell epitopes was limited to a small number of residues, these rare convergence points represent high-value immunological hotspots where humoral and cellular responses may be co-localized. The broader spatial separation of B-cell and T-cell epitopes, however, is consistent with established principles of antigen processing and presentation and does not diminish the overall immunogenic potential of the antigen.

Importantly, the convergence of structural conservation and dual-class epitope density distinguishes Zot from many previously proposed vaccine targets. While numerous candidate antigens exhibit either sequence conservation or predicted immunogenicity, Zot uniquely combines both features within a compact and structurally stable domain. This dual optimization—evolutionary constraint coupled with immunological accessibility—positions Zot as a particularly robust candidate for rational vaccine design.

From a translational perspective, these findings support a multi-epitope vaccine strategy centered on the conserved Antigen 2 domain. The identified epitope set provides broad HLA coverage, high predicted binding affinity, and structural stability, all of which are critical parameters for effective vaccine formulation. By focusing on conserved, functionally constrained regions, such a design minimizes the likelihood of immune escape while maximizing cross-strain coverage. This approach is particularly relevant for *A. baumannii*, where genetic diversity and rapid acquisition of resistance determinants complicate traditional therapeutic strategies (60, 141).

While these findings strongly support Zot as a candidate immunological target, it is essential to emphasize that this study is computational in nature. All results should therefore be interpreted as hypothesis-generating. Experimental validation—including peptide–HLA binding assays, T-cell activation studies, and *in vivo* models—is required to confirm immunogenicity and protective efficacy.

## Methods

### UniProt and NCBI genbank database mining for Zot homolog identification

To comprehensively characterize the phylogenetic distribution and evolutionary breadth of Zonula occludens toxin (Zot)–like proteins across Gram-negative bacterial (GNB) pathogens, we performed systematic database mining using both the UniProt Knowledgebase (UniProtKB) and the NCBI GenBank protein repository. These complementary resources were selected because UniProtKB provides highly curated protein sequences with functional annotation, while GenBank offers broad coverage of annotated and predicted protein sequences derived directly from genome submissions, including bacteriophage-encoded proteins that may be underrepresented in curated datasets. Initial searches were conducted using “*Acinetobacter baumannii* Zonula occludens toxin*”* as the query term in UniProtKB and NCBI GenBank (databases accessed on 1 November 2025). Collectively, these searches yielded 552 predicted protein entities annotated as Zot or Zot-like across bacterial and bacteriophage genomes. To ensure meaningful structural and functional comparability with the canonical *Vibrio cholerae* Zot protein, downstream analyses were restricted to full-length or near full-length homologs (≥345 amino acids). This length threshold was chosen to exclude truncated, fragmentary, or incomplete sequences that could bias comparative sequence, structural, and phylogenetic analyses. Application of these inclusion criteria resulted in a curated dataset comprising 58 unique protein entities, along with 4 species-specific bacteriophage-encoded Zot proteins, all of which were retained for subsequent analyses. Cross-referencing between UniProtKB and GenBank ensured removal of redundant entries while preserving unique sequence variants derived from distinct genomic or phage contexts.

### BLASTp analysis and heatmap generation

Protein homology analysis was performed using BLASTp (NCBI BLAST+), with the HUMC1 protein sequence used as the reference query against the NCBI non-redundant protein database. Default scoring parameters were applied, and only statistically significant hits with E-values ≤ 1 × 10^-^¹³ were retained to ensure inclusion of robust homologs (103). From the complete BLASTp output, 62 significant protein matches were selected for downstream analysis, encompassing both bacterial and bacteriophage-derived sequences. Redundant entries, partial sequences, and truncated proteins were excluded to ensure accurate comparison of full-length or near full-length homologs (142). For each retained hit, alignment metrics including percent sequence identity, query coverage, alignment length, bit score, E-value, and taxonomic annotation were extracted directly from the BLASTp results (143). Query coverage was calculated as the proportion of the HUMC1 sequence spanned by each alignment. These parameters were compiled into a structured data matrix in which individual homologs were represented as rows and alignment features as columns.

To facilitate comparative visualization of evolutionary relationships, percent identity values were stratified into biologically meaningful tiers corresponding to near-identical homologs (≥85% identity), moderately conserved homologs (35–45% identity), and deeply diverged homologs (≤30% identity), while query coverage values were retained as continuous variables (91, 144). Heatmaps were generated using standard data visualization workflows implemented in either Python (Seaborn/Matplotlib) or R (pheatmap/ComplexHeatmap), with percent identity encoded as a continuous color gradient and query coverage incorporated as an overlaid or secondary dimension. Rows were ordered by descending percent identity relative to the HUMC1 query, producing a tiered visual structure that separated closely related, intermediate, and deeply divergent homologs. Phage-associated sequences were annotated separately to distinguish bacterial and viral contributions to the protein family (109, 145). The resulting heatmap provided an integrated representation of sequence similarity, alignment completeness, and taxonomic origin, enabling visualization of the evolutionary architecture of the HUMC1 protein family in a reproducible and uniform manner.

### Comparative protein structure modeling using AlphaFold3

Protein structures of full-length Zot and the C-terminal Antigen 2 domain were predicted using the deep learning–based framework AlphaFold3 (116), selected for its high accuracy and ability to model proteins independent of close structural templates. Sequences from representative *A. baumannii* strains (HUMC1 and ATCC 19606) were analyzed, and Antigen 2 (residues ~265–355) was modeled separately to improve domain-level resolution. Predictions were generated using default settings, with top-ranked models selected based on confidence metrics.

Model quality was assessed using predicted Local Distance Difference Test (pLDDT) scores and Predicted Aligned Error (PAE). Regions with pLDDT ≥70 were considered reliable, with ≥90 indicating high confidence. PAE values were used to evaluate inter-domain relationships and potential flexibility, with low values (<5 Å) indicating well-defined structural regions. Comparative structural analysis between strains was performed using backbone superposition to assess conservation of overall fold and domain architecture.

Predicted B-cell and T-cell epitopes were mapped onto the AlphaFold3-derived structures to evaluate their spatial localization and potential surface accessibility. Regions with lower confidence scores were interpreted as potentially flexible, consistent with dynamic segments that may contribute to antibody recognition.

While AlphaFold3 provides high-confidence structural predictions, these models represent static conformations and do not capture full protein dynamics (117). Therefore, results should be interpreted as structural hypotheses, and further validation (e.g., molecular dynamics simulations or experimental studies) would be required to confirm stability and epitope accessibility under physiological conditions.

### Allele-resolved mapping of MHC class I and class II peptide–HLA binding landscapes

A comprehensive, allele-resolved mapping of peptide–MHC interactions was performed to characterize the intrinsic immunogenic architecture of the antigen across both MHC Class I and Class II presentation pathways. The full-length antigen sequence was computationally parsed into overlapping peptide libraries optimized for each pathway. For MHC Class I analysis, all possible 8–11 amino acid peptides were generated to capture canonical nonameric cores and extended variants compatible with HLA-A, HLA-B, and HLA-C binding grooves. For MHC Class II analysis, overlapping 15-mer peptides were generated using a one–amino-acid sliding window to ensure exhaustive coverage of potential CD4^+^ T-cell epitopes and to accommodate multiple binding registers within the open-ended MHC-II groove. Redundant peptides were removed to yield non-overlapping, non-redundant peptide sets for each class.

Allele-specific peptide–MHC binding affinities were predicted using the Immune Epitope Database (IEDB) NetMHCpan and NetMHCIIpan algorithms (146–149). MHC Class I binding predictions were performed using NetMHCpan (v4.x) across a representative panel of HLA-A, HLA-B, and HLA-C alleles, while MHC Class II binding predictions were generated using NetMHCIIpan (v4.x) across HLA-DR, HLA-DQ, and HLA-DP alleles, including common DRB1, DQA1/DQB1, and DPA1/DPB1 haplotypes. All predictions were reported as percentile ranks, which normalize predicted binding affinities relative to large, allele-specific reference peptide sets. Lower percentile values correspond to stronger predicted peptide–MHC binding and permit direct comparison across alleles with distinct binding repertoires. To preserve the full thermodynamic spectrum of peptide–HLA interactions, raw percentile ranks were retained without initial thresholding and visualized as continuous binding landscapes. Percentile values were min–max normalized within each panel and rendered using the viridis colormap, in which purple denotes weak predicted binding (high percentile ranks), green reflects intermediate affinity, and yellow indicates strong predicted binding (low percentile ranks). This unthresholded background enabled visualization of global allele-specific binding heterogeneity, structural constraints imposed by MHC binding grooves, and affinity gradients across the antigen, providing contextual separation between general binding permissiveness and high-confidence immunologically actionable epitopes.

Biologically relevant binders were subsequently defined using established IEDB (149) Analysis Resource (www.iedb.org) percentile thresholds. For MHC Class I, peptides with predicted binding percentiles ≤2% were classified as binders, and those with percentiles ≤0.5% were designated high-affinity binders, reflecting the stringent binding requirements imposed by the closed MHC-I groove and its reliance on canonical anchor residues. For MHC Class II, peptides with predicted binding percentiles ≤10% were classified as binders, and those with percentiles ≤2% were designated high-affinity binders, consistent with the broader permissiveness and register flexibility of the open MHC-II groove. These binder classifications were encoded as categorical overlays and superimposed onto the continuous viridis background to allow simultaneous interpretation of quantitative affinity landscapes and threshold-defined epitope significance. Allele-resolved peptide–MHC binding matrices were constructed separately for MHC Class I and MHC Class II, with peptides displayed along the y-axis and HLA alleles along the x-axis. Peptides that did not meet binder criteria for any allele were excluded from visualization to reduce background noise and emphasize immunologically relevant regions. Threshold-defined binders were overlaid using uniform color blocks, with standard binders indicated in pink and high-affinity binders indicated in red. This layered visualization strategy preserved both structural binding permissiveness and high-confidence epitope–HLA interactions within a single integrated framework.

To identify antigenic regions capable of engaging both antigen presentation pathways, peptide regions exhibiting overlapping binding signatures across MHC Class I and Class II heatmaps were examined for spatial concordance of binder overlays. Peptides or contiguous antigen regions demonstrating simultaneous MHC-I and MHC-II binding were designated as dual-class epitope regions, reflecting conserved sequence motifs capable of assuming multiple structural registers. These regions were prioritized due to their potential to support coordinated activation of CD8^+^ cytotoxic T lymphocytes and CD4^+^ helper T cells, a requirement for durable cellular immunity, memory formation, and effective humoral responses.

All heatmaps and overlays were generated using Python-based analytical pipelines (NumPy, Pandas, and Matplotlib). Visualization parameters, including font sizes, axis labeling, and legend placement, were optimized for clarity and publication-quality resolution. Allele ordering and peptide indexing were preserved across analyses to ensure reproducibility and facilitate direct comparison between MHC Class I and Class II binding landscapes.

### B cell methods (technical, algorithm- and parameter-specific version)

Comprehensive linear B-cell epitope prediction for Antigen 2 was performed using a multi-algorithm consensus workflow integrating seven independent computational predictors, each representing a distinct biochemical, structural, or machine-learning-derived criterion for antibody accessibility. All analyses were conducted using the IEDB (149) with default and experimentally validated parameter settings unless otherwise stated. Bepipred 2.0 predictions were run using the Random Forest–based model trained on epitope and non-epitope crystal structure–derived features, with scores exported at single-residue resolution and thresholded at the recommended 0.5 cutoff to identify linearly contiguous epitope segments (127).

Chou–Fasman β-turn propensity predictions were generated using the IEDB Chou–Fasman module (revision 2.2) with default conformational parameters (P(t), P(α), P(β), P(turn) values taken from empirically derived datasets), outputting residues with β-turn propensities >1.00 as structural hotspots indicative of loop regions prone to antibody recognition (128). Emini surface accessibility was calculated using the classical 6-residue sliding window method with solvent accessibility constants scaled to the default exposure threshold of 1.0, marking residues with scores above this value as exposed (129). Karplus–Schulz flexibility indices were computed by applying an empirically derived 7-residue sliding window using standard vibrational mobility constants, where residues with flexibility scores >1.05 were interpreted as dynamically mobile and thus more likely to form accessible B-cell epitopes (130).

Kolaskar–Tongaonkar antigenicity predictions were generated using physiochemical propensity scales derived from 156 experimentally validated epitopes, implementing a default antigenicity threshold of 1.00 to isolate enriched immunogenic stretches (131). Parker hydrophilicity values were computed using a 7-residue sliding window set to the canonical HPLC retention-time–based hydrophilicity scale, with residues scoring above 1.18 designated hydrophilic and therefore candidate epitope peaks (132). All heatmaps, amino-acid–position mappings, and epitope distribution visualizations were generated in Python 3.10 using NumPy (v1.26), Pandas (v2.1), and Matplotlib (v3.8) within a controlled Linux-based environment to ensure reproducibility.

For each predictor, per-residue scores were exported and aligned to the Antigen 2 primary amino acid sequence. Overlapping high-scoring segments across ≥4 independent algorithms were classified as “consensus B-cell epitopes.” Regions demonstrating complete or near-complete convergence across all seven algorithms were classified as “high-confidence epitope hot zones.” Each epitope’s boundaries were refined by tracing contiguous stretches of above-threshold residues and applying endpoint smoothing rules to remove single-residue anomalies. This process identified five major linear B-cell epitopes spanning positions 1–14, 18–32, 33–47, 50–66, and 69–90.

To generate positional heatmaps, each epitope was encoded as a binary vector across the antigen length (positions scored as 1 inside epitope boundaries and 0 outside). These binary maps were visualized as a stacked matrix using Matplotlib’s imshow function with a normalized viridis colormap. Sequence labels corresponding to the full peptide sequences (e.g., EKRNAELQAVNDVR) were plotted on the y-axis, and amino acid positions (1–90) were plotted on the x-axis. No assumption of statistical independence was made between prediction algorithms, and outputs were not combined using formal statistical aggregation. Instead, predictions were interpreted qualitatively based on cross-method agreement and normalized relative signal enrichment.

### High-stringency identification and characterization of shared MHC-I and MHC-II epitope cores

The target antigen sequence was analyzed for T-cell epitope content using NetMHCpan and NetMHCIIpan eluted-ligand (EL) prediction algorithms to identify peptides capable of binding MHC Class I and Class II molecules, respectively (121). MHC-I predictions generated 9-mer peptides with associated binding scores and percentile ranks, while MHC-II predictions produced variable-length peptides with defined 9-mer binding cores and percentile values. Prediction outputs were compiled into tabular datasets containing peptide sequence, predicted binding percentile, HLA allele, positional information, and algorithm-defined core sequences. To focus on the most immunologically relevant candidates, stringent percentile-based thresholds were applied independently to each dataset, retaining only peptides with a median predicted binding percentile ≤2% for MHC-I and ≤10% for MHC-II. These cutoffs were selected to enrich for high-affinity binders while preserving sufficient HLA diversity for broad population coverage.

Epitope cores were defined using the algorithm-assigned eluted-ligand core sequences for each class. Shared epitope cores were identified by strict intersection of the filtered MHC-I and MHC-II datasets, requiring exact identity of the 9-mer core sequence between classes. No allowance was made for partial overlap, shifted frames, or near matches, ensuring that retained epitopes represent peptides predicted to be processed and presented through both antigen-presentation pathways using the same core sequence. Using this conservative approach, a total of 13 shared epitope cores were identified, representing the most robust dual-class immunogenic elements within the antigen.

Quantitative analysis of these shared cores demonstrated strong and consistent predicted binding across both MHC classes. For MHC-I, binding percentiles for the shared cores ranged from 0.01% to approximately 1.8%, with the majority of core–allele interactions clustering below 0.5%, indicative of very high predicted affinity. Several shared cores exhibited ultra-low MHC-I percentile values (often 0.01–0.20%) across multiple HLA-A and HLA-B alleles, reflecting strong CD8^+^ T-cell presentation potential. In parallel, MHC-II binding percentiles for the same 13 cores ranged from ~0.14% to 9.8% across DR, DQ, and DP haplotypes, with several epitopes demonstrating particularly strong MHC Class II binding. Notably, MHC-II percentiles frequently showed below 2% for multiple DRB1 and DRB3 alleles, while mid-region cores displayed broader but still high-stringency MHC Class II binding, typically within the 3–8% range.

To visualize the breadth and strength of dual-class presentation, a shared-core binding matrix was constructed using the median predicted binding percentile for each epitope–allele pair, retaining the strongest value when multiple predictions existed for the same combination. The resulting heatmap displayed shared epitope cores as rows and HLA alleles as columns, encompassing both MHC-I and MHC-II alleles without normalization or scaling, thereby preserving direct quantitative interpretability. Across this matrix, the 13 shared cores collectively engaged 38 distinct HLA alleles, with individual epitopes binding between 3 and 15 total alleles. On average, each shared core interacted with 7–9 HLA alleles, demonstrating a high degree of redundancy in antigen presentation.

The shared cores were distributed across multiple conserved regions of the antigen, including the N-terminal region, the central NP–RPY and YEVTAKPVF-containing loops, and the C-terminal domain. This spatial distribution indicates that dual-class immunogenicity is not confined to a single hotspot but rather embedded throughout the protein. Importantly, several of the most broadly reactive cores combined ultra-high MHC-I affinity with strong or moderate MHC-II binding, a configuration known to support coordinated CD8^+^ cytotoxic and CD4^+^ helper T-cell responses. The convergence of high-affinity MHC-I and MHC-II binding onto this restricted set of conserved epitopes highlights a dense and overlapping T-cell epitope architecture, which is a defining feature of effective subunit vaccine antigens.

Overall, the integration of stringent prediction thresholds, exact shared-core matching, and quantitative binding analysis revealed a compact yet highly potent network of 13 shared epitope cores that dominate the antigen’s dual-class immunogenic landscape. These epitopes combine high predicted affinity, broad HLA coverage, and structural conservation, supporting their relevance for eliciting robust, durable, and population-wide T-cell–mediated immune responses.

## Data Availability

The datasets presented in this study can be found in online repositories. The names of the repository/repositories and accession number(s) can be found in the article/**Supplementary Material**.

## Supplementary material

Supplementary Table 1 High-resolution BLAST alignment analysis of the Antigen 1 (N-terminal) domain across Acinetobacter baumannii clinical isolates. Pairwise BLASTp alignments of the Antigen 1 (N-terminal) region of the Zonula occludens toxin (Zot) were conducted across a diverse panel of *Acinetobacter baumannii* strains, encompassing both reference genomes and clinically derived isolates. Quantitative alignment metrics—including bit score, percent identity, query coverage, alignment length, and E-values—demonstrate substantial sequence conservation across the dataset, with consistently high coverage and statistically significant homology. Compared to the C-terminal domain, the Antigen 1 region exhibits modestly increased sequence variability, with divergence localized to discrete residue positions, suggesting the presence of regions under relaxed evolutionary constraint or potential adaptive pressure. Despite this variability, the majority of the domain remains well conserved, indicating preservation of core structural features. Importantly, conserved segments within Antigen 1 overlap with predicted immunogenic regions, supporting their potential contribution to T-cell recognition. However, the presence of localized variability may influence epitope stability and cross-strain coverage. Collectively, these findings position the Antigen 1 domain as a moderately conserved antigenic region that may complement the highly conserved Antigen 2 domain in multi-epitope vaccine design strategies.

Supplementary Table 2 High-resolution BLAST alignment analysis of the Antigen 2 (C-terminal) domain across Acinetobacter baumannii clinical isolates. Pairwise BLASTp alignments of the Antigen 2 (C-terminal) region of the Zonula occludens toxin (Zot) were performed across a diverse panel of *Acinetobacter baumannii* strains, including reference and clinical isolates. Quantitative alignment metrics—bit score, percent identity, query coverage, alignment length, and E-values—demonstrate consistently high sequence conservation, with near-complete query coverage and uniformly low E-values indicative of strong homology across strains. The majority of sequences exhibit minimal divergence, with only sparse residue-level variability observed at discrete positions, suggesting pronounced evolutionary constraint within this domain. Notably, the high degree of conservation observed across phylogenetically distinct isolates reinforces the structural stability of the C-terminal region and aligns with its enrichment in predicted high-affinity MHC Class I and II binding epitopes. This convergence of sequence conservation and immunogenic potential supports the Antigen 2 domain as a prioritized target for broad-spectrum immunotherapeutic and vaccine design, with reduced likelihood of immune escape due to limited sequence variability.
